# TaCIPK10 interacts with and phosphorylates TaNH2 to activate wheat defense responses to stripe rust

**DOI:** 10.1111/pbi.13031

**Published:** 2018-12-05

**Authors:** Peng Liu, Jia Guo, Ruiming Zhang, Jiaxin Zhao, Cong Liu, Tuo Qi, Yinghui Duan, Zhensheng Kang, Jun Guo

**Affiliations:** ^1^ State Key Laboratory of Crop Stress Biology for Arid Areas College of Plant Protection Northwest A&F University Yangling Shaanxi China

**Keywords:** wheat, TaCIPK10, resistance, phosphorylation, TaNH2, *Puccinia striiformis* f. sp*. tritici*

## Abstract

Calcineurin B‐like interacting protein kinase (CIPKs) has been shown to be required for biotic stress tolerance of plants in plant‐pathogen interactions. However, the roles of CIPKs in immune signalling of cereal crops and an in‐depth knowledge of substrates of CIPKs in response to biotic stress are under debate. In this study, we identified and cloned a CIPK homologue gene *TaCIPK10* from wheat. *TaCIPK10* was rapidly induced by *Puccinia striiformis* f. sp*. tritici* (*Pst*) inoculation and salicylic acid (SA) treatment. *In vitro* phosphorylation assay demonstrated that the kinase activity of TaCIPK10 is regulated by Ca^2+^ and TaCBL4. Knockdown *TaCIPK10* significantly reduced wheat resistance to *Pst*, whereas *TaCIPK10* overexpression resulted in enhanced wheat resistance to *Pst* by the induction of defense response in different aspects, including hypersensitive cell death, ROS accumulation and pathogenesis‐relative genes expression. Moreover, TaCIPK10 physically interacted with and phosphorylated TaNH2, which was homologous to AtNPR3/4. Silencing of *TaNH2* in wheat resulted in enhanced susceptibility to the avirulent *Pst* race, CYR23, indicating its positive role in wheat resistance. Our results demonstrate that TaCIPK10 positively regulate wheat resistance to *Pst* as molecular links between of Ca^2+^ and downstream components of defense response and TaCIPK10 interacts with and phosphorylates TaNH2 to regulate wheat resistance to *Pst*.

## Introduction

Wheat stripe rust, caused by *Puccinia striiformis* f.sp. *tritici* (*Pst*), an obligate biotrophic pathogen, poses a tremendous threat to the production of wheat worldwide (Wan *et al*., 2004). Two strategies are used to control rust in wheat: chemical control and genetic control. Genetic control is the most cost‐effective strategy to reduce the threat of the disease because of its environmental and economic factors (Ellis *et al*., 2014). Normally, two general classes of genes based on their phenotypic effects are used for genetic control: pathogen race‐ or strain‐specific resistance (R genes) and adult plant resistance (APR) genes (Chen, 2005). R genes usually mediate resistance during all plant developmental stages, whereas APR genes function mainly during the adult stage. A series of defense responses have been confirmed to be involved in wheat all‐stage resistance against *Pst* infection, including cell wall apposition, papilla formation and accumulation of several antifungal compounds, such as lignin, calloses, plant hydrolases and reactive oxygen species (ROS; Wang *et al*., 2007; Zhang *et al*., 2008). In addition, many permanently or temporarily named *Yr* genes and quantitative trait loci (QTL) have been identified to confer APR to stripe rust (Zhou *et al*., 2014; Lu *et al*., 2014). Besides these two classes of genes, numerous genes in wheat have been extensively reported to be involved in the interaction between wheat and *Pst* (Kang *et al*., 2017). However, as an obligate biotrophic basidiomycete, *Pst* cannot be cultured *in vitro*. Consequently, studies on the mechanism of wheat resistance to *Pst* are challenging.

Normally, plants establish a series of immune events in response to pathogen infection, including ion fluxes across the plasma membrane, an increase in the intracellular Ca^2+^ concentration, accumulation of ROS, biosynthesis of salicylic acid (SA), cell‐wall strengthening, etc. (Boller and Felix, 2009; Tsuda and Katagiri, 2010). Among these signalling processes, SA has been demonstrated to be a critical modulator of multiple levels of immunity, protecting plants from a wide range of phytopathogens by mediating immune responses at both local and systemic levels (Vlot *et al*., 2009). The mechanism of the mode of action of SA has been studied in‐depth in model plant *Arabidopsis*. SA signalling through NPR1 (nonexpresser of PR protein 1) is required to establish systemic acquired resistance (SAR). In the absence of pathogen attack, NPR1 is present in the cytoplasm as disulfide‐bound oligomers. NPR3 and NPR4, the paralogues of NPR1, directly bind SA and function as substrate adaptors that regulate proteasome‐mediated degradation of NPR1 (Fu *et al*., 2012). However, the research on mechanism of NPR1 homologue genes is still very shallow. In rice, overexpression of the rice *NPR1* homologues *NH1* and *NH3* result in enhanced resistance to *Xanthomonas oryzae* pv. *oryzae* (*Xoo*) and *Magnaporthe oryzae* (Yuan *et al*., 2007; Bai *et al*., 2011), suggesting that *OsNH1* and *OsNH3* play a positive role in rice resistance. Another NPR1‐like gene, *OsNPR2*/*NH2*, was shown to response to infection by the bacterial blight pathogen, *Xoo,* and the rice blast fungus, *M. oryzae* (Yuan *et al*., 2007).

SA‐mediated plant immunity has been confirmed to be regulated by calcium signals. In *Arabidopsis*, the calmodulin‐binding transcription activator CAMTA3 (also called AtSR1) directly binds the promoter of EDS1 to repress the expression of *EDS1*, which is known to be required for NLR‐mediated cell death and activation of SA biosynthesis (Yuan *et al*., 2018). Calcium functions as a second messenger and regulates a plethora of inter‐ and extracellular signalling processes. The transient changes in Ca^2+^ concentration is sensed and decoded by sensor relay and responder proteins, such as calmodulins (CaMs), calcium‐dependent protein kinases (CDPK or CPKs) and calcineurin B‐like proteins (CBLs). Unlike CaM and CDPK, which interact with a large number of target proteins, CBLs specifically interact with a family of protein kinases referred to as CBL‐interacting protein kinases (CIPKs), which belong to the Snf1‐RELATED KINASE3 family (Suc nonfermenting 1‐related kinases, group 3; SnRK3). It has been shown that the protein kinase of CIPKs is regulated by CBLs binding Ca^2+^ through their four EF hands (Halfter *et al*., 2000; Kurusu *et al*., 2010). Bioinformatic analyses identified ten CBLs and 26 CIPKs in *Arabidopsis* and seven CBLs and 32 CIPKs in wheat (Hashimoto *et al*., 2012; Sun *et al*., 2015). Different CBLs interact specifically with different CIPKs, resulting in a vast array of potential CBL/CIPK module combinations and versatility of Ca^2+^ responsiveness to this signalling system. Each potential CBL/CIPK module generates specificity in the signalling pathway and functions for crosstalk or overlap in signalling pathways (Thoday‐Kennedy *et al*., 2015). Most CBL‐CIPK components have been thoroughly studied in the regulation of different abiotic stress‐triggered signalling pathways (Cheong *et al*., 2003; Guo *et al*., 2002; Pandey *et al*., 2004, 2005), but the function of the CBL‐CIPK system in response to biotic stresses is still an under‐studied area of research. In *Arabidopsis*, NPR1 is phosphorylated and activated by PKS5/CIPK11 to induce *WRKY38* and *WRKY62* after bacterial inoculation, suggesting that crosstalk occurs between CBL/CIPK modules and SA signalling pathways during biotic stress responses (Xie *et al*., 2010). To date, the relationship of Ca^2+^ signals and *NPR1*‐like genes in stress responses in crop plants is still unclear. Therefore, characterization of *CIPK* and *NPR1*‐like genes in wheat is a worthwhile goal, especially towards elucidating mechanisms of pathogen resistance.

In this study, we identified and functionally characterized one *CIPK* gene, *TaCIPK10*, and assessed its important roles in wheat resistance to *Pst*. Additionally, we demonstrated that TaCIPK10 kinase activity is regulated by conjoined TaCBL4 protein and Ca^2+^. Concurrently, TaNH2, an *Arabidopsis* NPR3/4 orthologues, which was confirmed to interact with and be phosphorylated by TaCIPK10, plays a positive role in wheat resistance. Our results demonstrated that TaCIPK10 transmit the Ca^2+^ signals to positively regulate wheat resistance to *Pst* and TaNH2 may functions as target of TaCIPK10 in wheat response to *Pst* infection. Our results provide new insights towards understanding the roles of CBL‐CIPK and the NPR family in plant resistance.

## Results

### *TaCIPK10* is significantly induced by *Pst* infection and SA treatment

**Figure 1:**
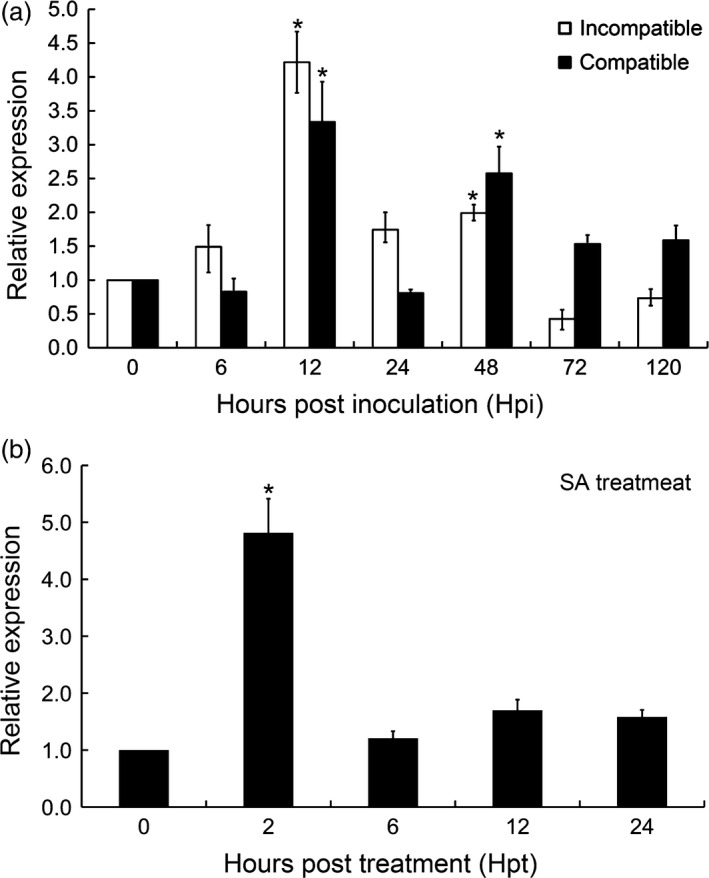
Transcript profiles of *TaCIPK10* in wheat leaves in response to *Pst* infection and SA treatment. (a) *TaCIPK10* is induced by *Pst* infection. Wheat leaves inoculated with *Pst* isolates CYR23 (incompatible interaction) and CYR31 (compatible interaction) were sampled at 0, 6, 12, 24, 48, 72 and 120 h postinoculation (hpi). (b) SA treatment enhances *TaCIPK10* expression. Wheat seedlings were treated with 2 mm SA and collected at 0, 2, 6, 12 and 24 h posttreatment (hpt). Wheat *TaEF‐1α* was used as reference. The un‐inoculated plants at time 0 was used as control. Relative transcript level of *TaCIPK10* was calculated by the comparative threshold (2^−ΔΔCT^) method. The quantitative RT‐PCR values were normalized to those for *TaEF‐1α* and are presented as fold changes relative to that in un‐inoculated plants at time 0. The transcript level of *TaCIPK10* in the wheat leaves at time 0 was standardized as 1. Asterisks indicate significant differences between time‐course points using Student's *t*‐test (*P* < 0.01).

A total of 32 *TaCIPK* genes were identified in the wheat genome database (Sun *et al*., 2015). In rice, OsCIPK14/15 was confirmed to be involved in plant immunity (Kurusu *et al*., 2010). To investigate whether CIPKs participate in wheat responses to *Pst*, the transcript levels of four *TaCIPK* genes (*TaCIPK2*,* 10*,* 14*,* 15*), which located in the same cluster with OsCIPK14/15 (Sun *et al*., 2015), were determined by qRT‐PCR in leaves of wheat seedlings following *Pst* infection. The results showed that transcript levels of all selected *TaCIPKs* were affected by *Pst* infection (*P* < 0.01 and fold change >2; Figure 1a and Figure S1), suggesting that *TaCIPKs* participate in the interaction between wheat and *Pst* (Figure S1).

As shown in previous studies, the SA‐mediated defense signalling pathway is activated following infection by biotrophic pathogens (Vlot *et al*., 2009). Thus, we measured SA concentrations during wheat resistance to *Pst*. The results indicated that SA concentrations were significantly increased during the interaction between wheat and *Pst*, suggesting that the SA signalling pathway is involved in wheat resistance to *Pst* (Figure S2). In order to explore whether the mechanisms of *TaCIPKs* in wheat resistance are related to SA, transcript levels of these *TaCIPKs* were determined in leaves of wheat seedlings following exogenous application of SA. The results showed that transcript levels of *TaCIPK10* are significantly enhanced fivefold at 2 hpt (Figure 1b), while those of other *CIPKs* were not significantly induced (Figure S1). Based on these observations, *TaCIPK10* was selected for further analysis during the response of wheat to *Pst* infection.

### Sequence analysis and subcellular localization of TaCIPK10

According to the sequences identified in the wheat genome, the full‐length cDNA of *TaCIPK10*, encoding a putative protein composed of 438 amino acid residues with a molecular weight of 49.9 kDa and an isoelectric point (pI) of 9.0, was obtained from wheat cv. Suwon11 by RT‐PCR. BlastN analysis of the wheat genome identified three copies of *TaCIPK10* with 96% nucleotide sequence identity, which are located on chromosomes 4A, 4B and 4D (Figure S3a). TaCIPK10 obtained from Suwon11 was on chromosome 4D and contained a few amino acid variations relative to other predicted amino acid sequences on chromosome 4A and 4B (Figure S3b). *In silico* sequence analysis revealed that TaCIPK10 contains a kinase catalytic domain and a regulatory domain. The activation loop containing three conserved phosphorylatable residues (threonine, serine and tyrosine) were found in the kinase catalytic domain, while the NAF motif (also known as FISL motif), mediated CIPK binding to CBL, in the regulatory domain (Figure S4a). Based on the amino acid sequences of CIPKs with assigned functions, TaCIPKL10 shared the same clade with other CIPK10 protein sequences from monocot plants (Figure S4b). In addition, the GFP fusion protein of TaCIPK10 was mainly distributed in the cytoplasm of wheat protoplasts (Figure S5).

### TaCIPK10 interacts with several TaCBLs

**Figure 2:**
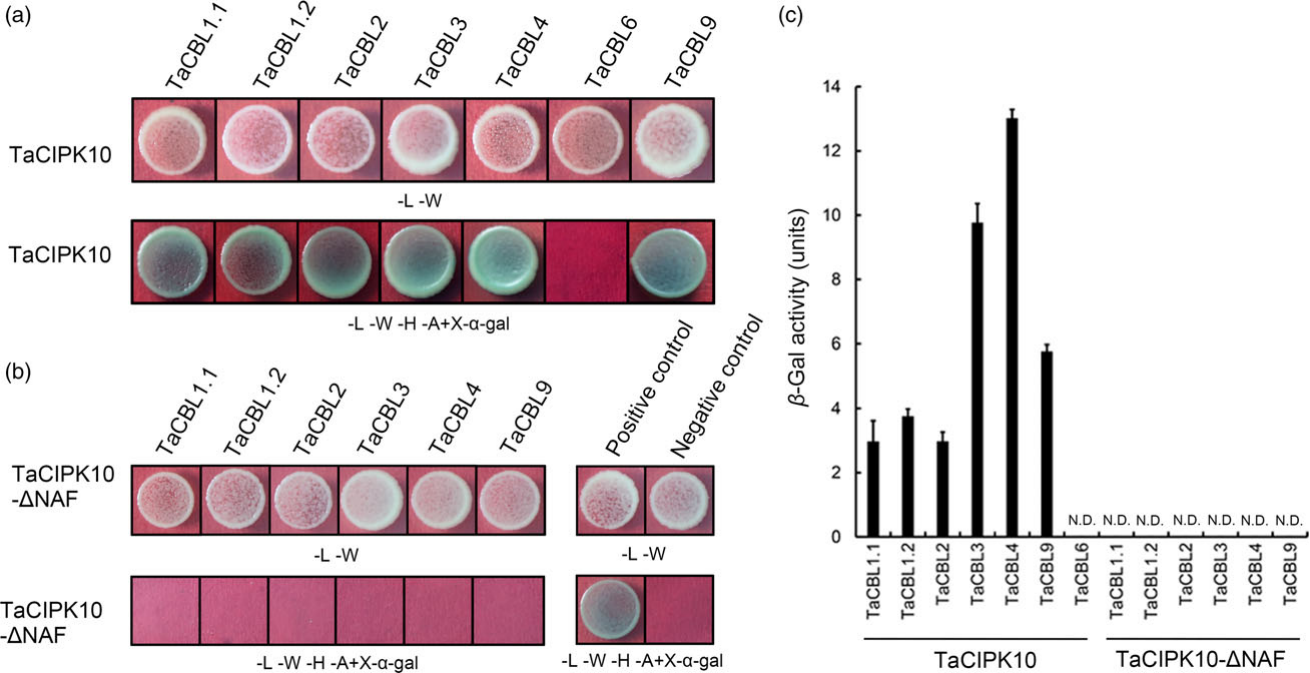
TaCIPK10 interacts with TaCBLs in a yeast two‐hybrid system. (a, b) TaCIPK10 (a) or TaCIPK10Δ (b) was cloned into pGBKT7 vector and TaCBLs were cloned into the pGADT7 vector. Cells of yeast strain AH109 harbouring the indicated plasmid combinations were grown on either the nonselective (SD‐LW) or selective (SD‐LWHA) medium containing 20 μg/mL X‐α‐gal. Positive control, the interaction between SV40 large T‐antigen (T) and murine p53 (53) T‐AD+53‐BD. Negative control, the interaction between SV40 large T‐antigen (T) and human lamin C (Lam), T‐AD+Lam‐BD. (c) Quantitative *β*‐galactosidase activity of combinations between TaCIPK10 and TaCBLs or TaCIPK10Δ and TaCBLs. N.D., Not detected. Data represent the mean of three biological replicates ±SE.

Seven CBL homologues in wheat cv. Suwon11 were investigated for interaction preferences with TaCIPK10 by the yeast two‐hybrid (Y2H) system. The results showed that TaCIPK10 interacts with six TaCBLs (TaCBL1.1, TaCBL1.2, TaCBL2, TaCBL3, TaCBL4 and TaCBL9) but not with TaCBL6 (Figure 2a). The NAF motif in the CIPK family had been reported to interact with CBLs and possess auto‐inhibitory function (Pandey *et al*., 2014). As expected, TaCIPK10‐ΔNAF, a mutant protein deficient in the FISL/NAF motif, failed to interact with TaCBLs in yeast (Figure 2b), suggesting the highly conserved functions of FISL/NAF motif in CIPKs among plant species. The strongest interaction of TaCIPK10 with TaCBL4 was confirmed by *β*‐galactosidase activity (Figure 2c). In addition, protein expression in the Y2H system was confirmed by Western blot using Myc‐ and HA‐antibodies (Figure S6).

### The protein kinase activity of TaCIPK10 is regulated by TaCBL4 and Ca^2+^

**Figure 3:**
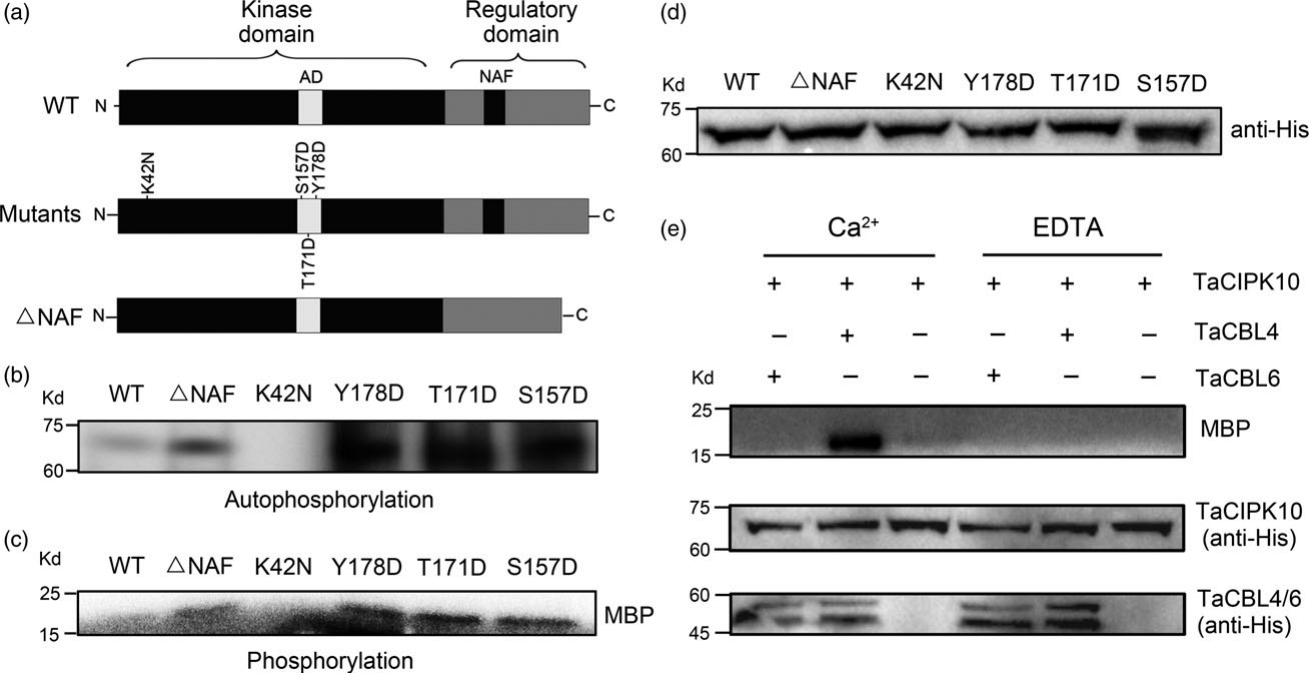
TaCIPK10 is an active kinase and regulated by TaCBL4 and Ca^2+^. (a) Schematic diagrams of TaCIPK10 mutants and deletions. AD, activation domain. NAF, the motif for interacting with CBLs. Mutagenized residues and deleted domain are marked. WT, the full length of TaCIPK10. Single amino acid was substituted for Lysine‐42 (K42) to Asparagine (N) and Serine‐157 (S157), Threonine‐171 (Y171), Tyrosine‐173 (Y173) to Aspartic acid (D) and designated as K42N, S157D, Y171D and Y173D, respectively. ΔNAF, NAF (amino acid 309–333) deletion. (b) For the autophosphorylation assays, TaCIPK10 and mutant versions were incubated with [γ‐^32^P] ATP and kinase buffer. The reaction products were analysed by SDS‐PAGE gels and autoradiography. (c) For the phosphorylation assays, the proteins were incubated with 3 μg MBP in the presence of [γ‐^32^P] ATP and kinase buffer. (d) Equimolecular levels of TaCIPK10 and mutant versions were confirmed by Western blot using anti‐His antibodies. (e) The kinase activity of TaCIPK10 is regulated by TaCBL4 in a Ca^2+^‐dependent manner. TaCIPK10 was incubated with MBP, [γ‐^32^P] ATP and Ca^2+^ or EDTA in kinase buffer in presence or absence of TaCBL4‐His and TaCBL6‐His fusion proteins.

As a Ser/Thr protein kinase, the kinase activity of TaCIPK10 was characterized. Three site‐directed mutants of TaCIPK10 were generated following previous studies (Pandey *et al*., 2014) of other CIPKs that conferred enhanced kinase activity (Figure 3a). A TaCIPK10 mutant version lacking the FISL/NAF motif was generated (as that in the experiment of TaCIPK10 interacts with several TaCBLs; Figure 3a). In addition, an inactive version, mutating a conserved lysine (K) to asparagine (N) at position 42, was generated as a negative control. As shown in Figure 3b,c, the autophosphorylation and phosphorylation activity of wild‐type TaCIPK10 was almost undetectable, whereas the other TaCIPK10 variants showed significantly increased kinase and autophosphorylation activity compared to the wild type. TaCIPK10‐K42N had no activity (Figure 3b,c). In addition, TaCIPK10 and its mutants with expected molecular sizes were detected by Western blot using anti‐His antibody (Figure 3d). To test if TaCIPK10 kinase activity was regulated by Ca^2+^ and TaCBLs, *in vitro* transphosphorylation assays were performed with MBP as substrate in the presence or absence of Ca^2+^. TaCBL4 and TaCBL6 were selected as the regulator of TaCIPK10 kinase activity because TaCIPK10 strongly interacted with TaCBL4 but not with TaCBL6. As shown in Figure 3e, the kinase activity of TaCIPK10 was significantly increased only in the presence of TaCBL4 and Ca^2+^, suggesting that the kinase activity of TaCIPK10 is modulated by TaCBLs in a Ca^2+^‐dependent manner.

### Suppression of *TaCIPK10* reduces wheat resistance to *Pst*

**Figure 4:**
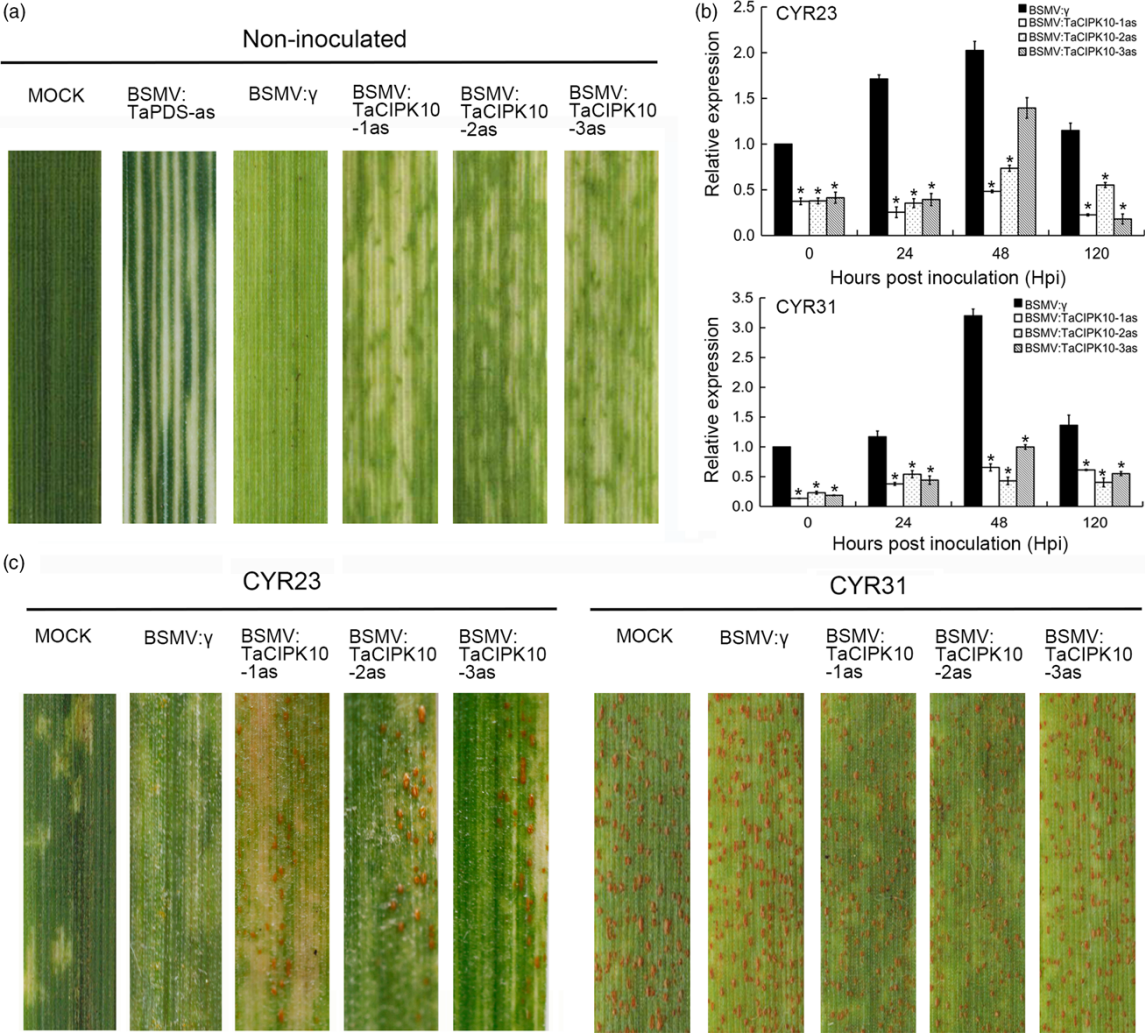
Silencing *TaCIPK10* reduces wheat resistance against *Pst* race CYR23. (a) Mild chlorotic mosaic symptoms were observed at 10 dpi on leaves inoculated with BSMV:γ, BSMV:TaPDS‐as and BSMV:TaCIPK10‐1/2/3as. Mock, wheat leaves treated with 1 × Fes buffer. (b) Relative expression of *TaCIPK10* during the interaction between *TaCIPK10*‐knockdown plants and CYR23 or CYR31. The *TaEF‐1α* gene was used for normalization. The quantitative RT‐PCR values were normalized to those for *TaEF‐1α*, and are presented as fold changes relative to that in plants with BSMV:γ treatment at time 0. The transcript level of *TaCIPK10* after *Pst* inoculation in the wheat leaves treated with BSMV:γ at time 0 was standardized as 1. Asterisks indicate significant differences between that in *TaNH2*‐knockdown plants and control plants at the same time points using Student's *t*‐test (*P* < 0.01). (c) Photos of fourth leaves of *TaCIPK10*‐knockdown plants inoculated with urediospores of the avirulent race CYR23 or the virulent race CYR31. Typical leaves were photographed at 15 dpi.

To characterize the function of *TaCIPK10* in wheat defense response to *Pst*, BSMV‐VIGS, a rapid and effective approach for analysing gene function in wheat and barley (Scofield *et al*., 2005), was used. Three specific fragments of *TaCIPK10* cDNA were selected for the silencing experiment (Figure S3a). The mild chlorotic mosaic symptoms appeared on BSMV‐inoculated plants, and BSMV:TaPDS‐as infected plants exhibited strong photobleaching at 10 dpi (Figure 4a), indicating that the BSMV‐mediated gene silencing system functioned correctly. The fourth leaf of all wheat plants was then inoculated with fresh urediospores of *Pst* race CYR23 or CYR31. The transcript level of *TaCIPK10* in silencing plants was much lower than that in control plants during the interaction between TaCIPK10‐knockdown plants and *Pst* (*P* < 0.01 and fold change <0.5), suggesting that TaCIPK10 was silenced successfully by BSMV system (Figure 4b). The transcript accumulation of each copy on chromosomes 4A, 4B and 4D could not be determined by qRT‐PCR because of high similarity and identity among them (Figure S3a). Meanwhile, sequence alignment among three TaCIPKs and TaCIPK10 which located in the same clade did not contain consecutive 21‐ to 24‐nucleotide sequences, indicating the specificity of virus‐induced gene silencing (VIGS) fragments used in this study (Figure S7). At 14 dpi with *Pst*, conspicuous HR symptoms appeared on all leaves after inoculation with the incompatible strain, CYR23 (Figure 4c). A few fungal uredia surrounding the necrotic areas were observed on leaves that were pre‐infected with BSMV:TaCIPK10‐1/2/3as (Figure 4c). In contrast, all leaves infected with the compatible strain, CYR31, exhibited normal disease development (Figure 4c). To determine whether transcript accumulation of defense‐related genes was affected by *TaCIPK10*, the transcript levels of three pathogenesis‐related (PR) genes and three ROS‐related genes were measured. The expression of *TaPR1*,* TaPR2* and *TaPR5* was significantly reduced during the interaction between *TaCIPK10*‐knockdown plants and CYR23 (Figure S8). Two ROS‐scavenging genes, *TaCAT* and *TaSOD*, were significantly increased in all *TaCIPK10*‐knockdown plants challenged by CYR23, while one ROS‐generating gene, *TaNOX*, was down‐regulated (Figure S8).

To confirm the host response in *TaCIPK10*‐knockdown plants after inoculation with *Pst* CYR23, the fourth leaves were analysed microscopically. The necrotic area per infection site was measured by autofluorescence, and DAB staining per infection site was observed for H_2_O_2_ accumulation (Figure S9a). The results showed that the area of H_2_O_2_ accumulation in all *TaCIPK10*‐knockdown plants was significantly lower than that in BSMV:γ infected leaves at 48 and 120 hpi but not at 24 hpi (Figure S9b), and the necrotic area was significantly decreased in *TaCIPK10* silenced plants compared to the control at 120 hpi (Figure S9c). In addition, haustoria, hyphal length and infection area of CYR23 in *TaCIPK10*‐knockdown plants were analysed by WGA staining and examined by histological observation (Figure S10a). The hyphal length and the number of haustoria in all *TaCIPK10*‐silenced leaves were comparable to those in BSMV:γ infected plants at 24 and 48 hpi (Figure S10b,c). However, the number of haustorial mother cells in leaves of *TaCIPK10*‐knockdown plants was significantly increased over that in BSMV:γ infected plants (Figure S10d), as was infection area at 120 hpi (Figure S10e). Taken together, the results indicate that silencing *TaCIPK10* diminishes the wheat defense response against *Pst*.

### Overexpression of *TaCIPK10* enhances wheat resistance to *Pst*

**Figure 5:**
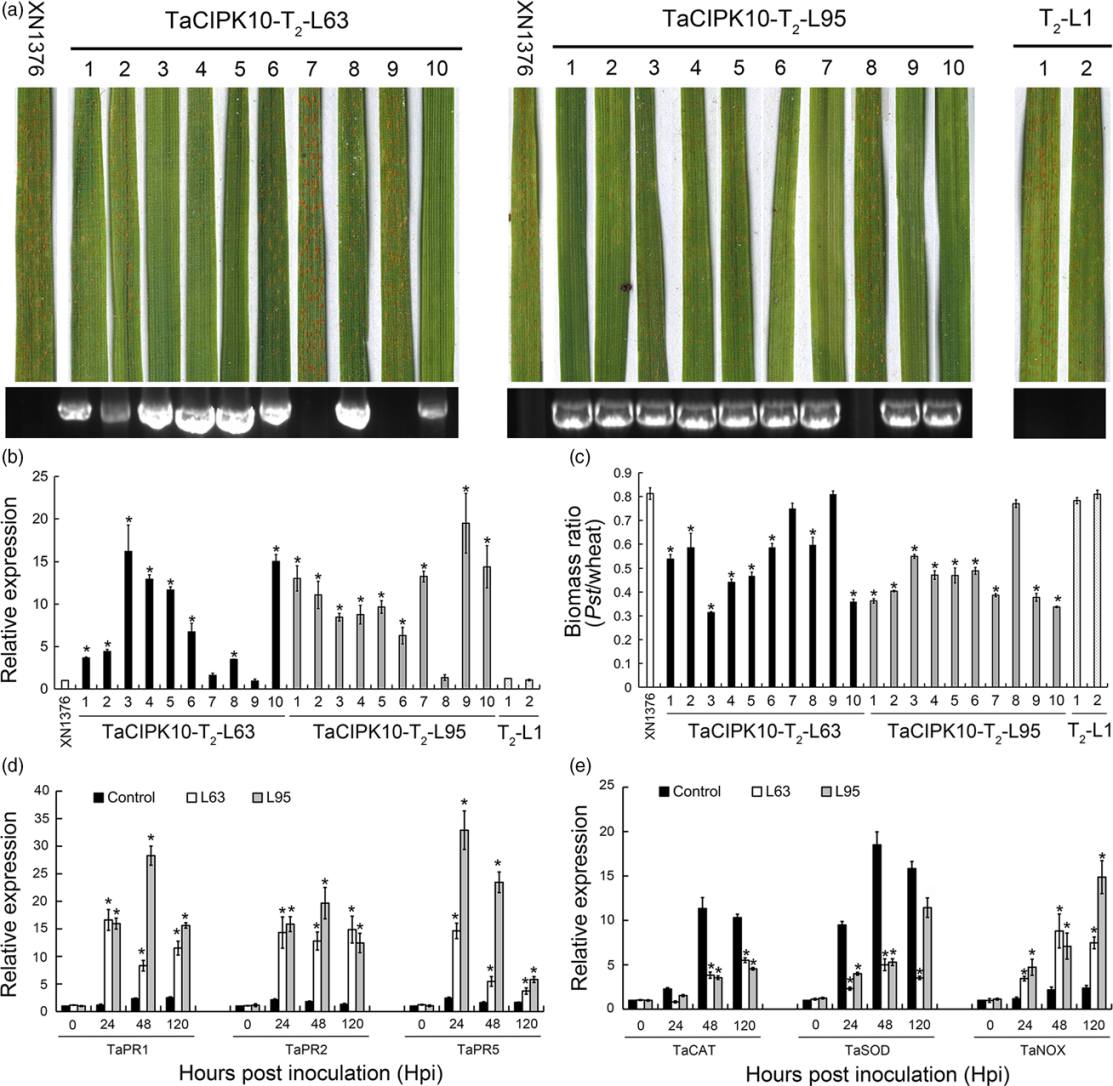
Overexpression of *TaCIPK10* enhances wheat resistance to *Pst*. (a) Foliar parts of lines TaCIPK10‐T_2_‐L63 and TaCIPK10‐T_2_‐L95 inoculated with avirulent *Pst* isolate CYR32. (b) Relative transcript levels of *TaCIPK10* in the leaves of lines TaCIPK10‐T_2_‐L63 and TaCIPK10‐T_2_‐L95. The data were normalized to those for *TaEF‐1a* and presented as fold changes relative to XN1376. Line TaCIPK10‐T_2_‐L1 was used as negative control. The transcript level of *TaCIPK10* in non‐transformed plants was standardized as 1. (c) Fungal and wheat biomass ratio measured via total DNA content at 14 dpi by absolute quantification using the internal reference genes *PsEF* and *TaEF*, respectively. (d, e) Relative transcript levels of *TaPR1*,* TaPR2*,* TaPR5*,* TaSOD*,* TaCAT* and *TaNOX* in *TaCIPK10* overexpression lines TaCIPK10‐T_2_‐L63 and TaCIPK10‐T_2_‐L95 inoculated with CYR32. The wild type was used as control. The transcript level of genes in control plants at time 0 was standardized as 1. Data represent the mean of three biological replicates ±SE. Asterisks indicate significant differences between that in transformed plants and control at the same time points using Student's *t*‐test (*P* < 0.01).

To further confirm the defense role of *TaCIPK10* in wheat, overexpression transgenic wheat plants were produced by co‐bombardment with *TaCIPK10* expression vector pWMB003‐TaCIPK10 and pAHC20 into 2000 immature embryos of the wheat cultivar Xinong1376 (XN1376). Twelve positive transgenic plants were identified from 12 regenerated T_0_ plants by PCR using specific primers (Figure S11a,b). The expression level of *TaCIPK10* was up‐regulated in all positive T_0_ generation plant compared to wild type and the negative plants (Figure S11c). Two lines with the highest expression level, TaCIPK10‐T_0_‐63 and TaCIPK10‐T_0_‐95, were selected for further experiments. Two T_1_ lines, TaCIPK10‐T_1_‐63 and TaCIPK10‐T_1_‐95, derived from relevant T_0_ generations, expressed enhanced resistance to *Pst* race CYR32 (Figure S11d,e), with an induction of *TaCPK10* transcription as high as 10‐fold compared to wild type (Figure S11e,g). At T_2_ generation, eight and nine positive plants from TaCIPK10‐T_2_‐63 and TaCIPK10‐T_2_‐95, respectively, were identified by PCR and qRT‐PCR (Figure 5a,b). The transcript accumulation of *TaCIPK10* was 3.4–19.4‐fold higher than that in XN1376 (Figure 5b). Fourteen days after *Pst* inoculation, all positive plants expressed increased resistance with a significant reduction in sporulation compared to wild type and negative plants. By applying the McNeal's uniform scale system (McNeal *et al*., 1971), we observed that the host response of wild type and negative control inoculated with *Pst* ranged from 7 to 8, showing abundant sporulation with few necrotic/chlorotic stripes and indicating high susceptibility of wheat. By contrast, the response of transgenic plants was in the 1–4 range, indicating high or medium resistance (Table S1). The biomass analysis showed that the biomass of *Pst* in positive plants was significantly reduced by 30%–65% (Figure 5c). In addition, the *PR* genes (*TaPR1*,* TaPR2* and *TaPR5*) and *TaNOX* showed higher expression levels than that in the wild type, while the *TaCAT* and *TaSOD* were down‐regulated during the interaction between two positive lines and CYR32 (Figure 5d,e). Overexpression of *TaCIPK10* stimulates wheat resistance response expressed as induction of necrotic area, increased H_2_O_2_ accumulation and decreased infection area of *Pst* at 14 hpi compared to the control (Figure S12). Moreover, the growth of *TaCIPK10* overexpression transgenic lines showed no obvious difference relative to control plants (Figure S13). These results indicate that elevated expression of *TaCIPK10* induces the accumulation of defense‐related gene transcripts, which enhanced the wheat resistance to *Pst*.

### TaCIPK10 interacts with and phosphorylates an *Arabidopsis* NPR3/4 orthologue protein in wheat

**Figure 6:**
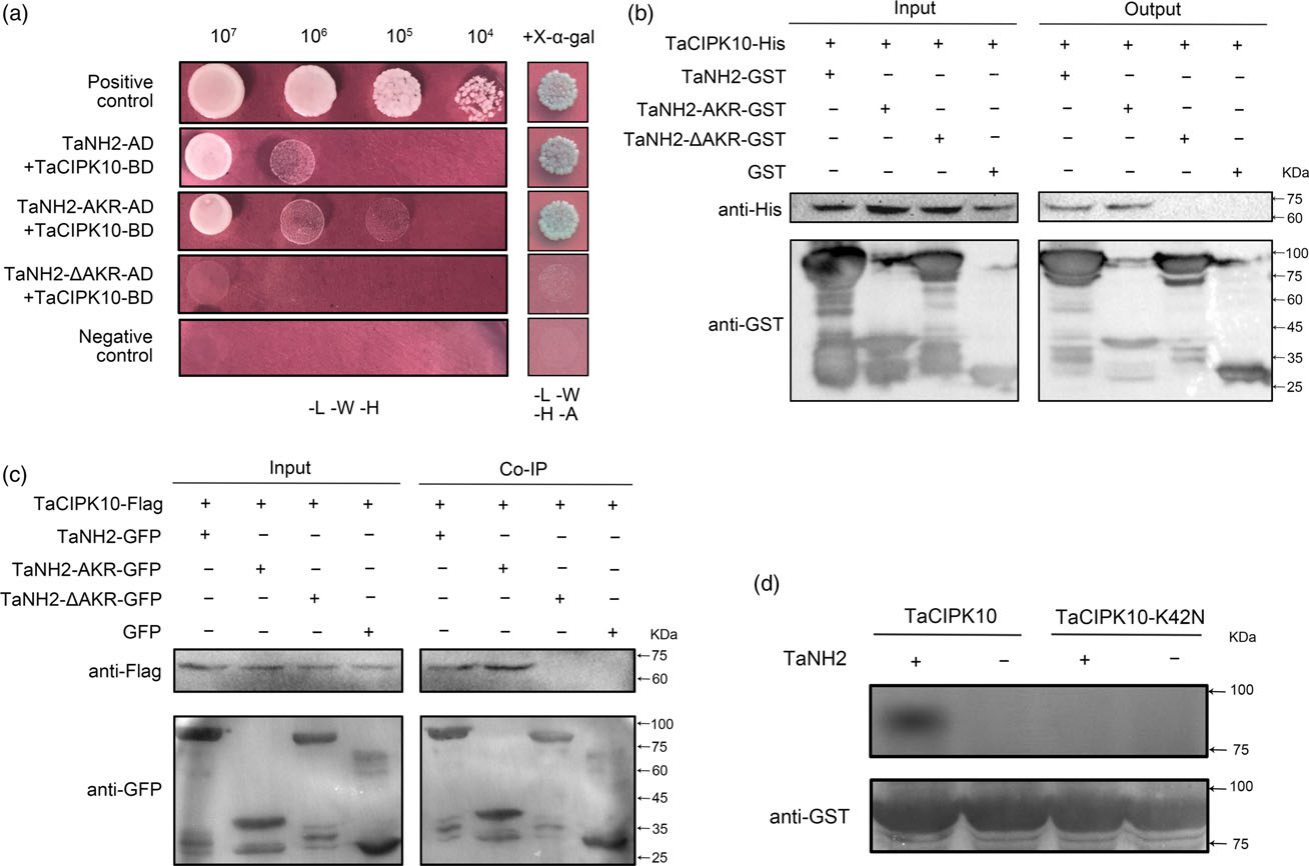
TaCIPK10 physically interacts with and phosphorylates TaNH2. (a) Yeast two‐hybrid analysis of interactions between TaCIPK10 and TaCNH2 or its derivatives. Cells of yeast strain AH109 harbouring the indicated plasmid combinations were cultivated on the selection medium (SD‐LWH and SD‐LWHA containing 20 μg/mL X‐α‐gal). Positive control, the interaction between SV40 large T‐antigen (T) and murine p53 (53) T‐AD+53‐BD. Negative control, the interaction between SV40 large T‐antigen (T) and human lamin C (Lam), T‐AD+Lam‐BD. (b) GST pulldown assay was used to detect the interaction between TaCIPK10‐His and TaNH2‐GST, TaNH2‐AKR‐GST, TaNH2(Δ)‐GST, or GST. The pulled down samples and the inputs were analysed by SDS‐PAGE and detected by Western blot using anti‐His antibody and anti‐GST antibody. (c) Protein interaction of TaCIPK10‐Flag and TaNH2‐GFP or its derivatives were determined by Co‐IP assay. The constructs of TaCIPK10‐Flag and TaNH2‐GFP or its derivatives tagged GFP were co‐expressed in *Nicotiana benthamiana*. GFP‐tagged protein and Flag‐tagged protein were isolated at 48 h after inoculation and analysed by anti‐GFP antibody followed by immunoblotting with anti‐FLAG antibodies. (d) TaNH2 is phosphorylated by TaCIPK10 *in vitro*. His‐tagged TaCIPK10 or its mutant (TaCIPK10‐k42N) was inoculated with recombinant TaNH2‐GST in reaction buffer in the presence of [γ‐^32^P] ATP, Ca^2+^ and TaCBL4. Proteins were subjected to SDS–PAGE followed by Western blotting using anti‐His and anti‐GST antibodies.

AtNPR1, a key regulator in the SA signalling pathway, was confirmed as the target of AtCIPK11 (AtPKS5) in a previous study (Xie *et al*., 2010), suggesting that CIPK families are related to NPR1 homologue. Therefore, we speculated that the function of TaCIPK10 resistance to *Pst* is related to the homologue of AtNPR1 in wheat. To confirm our speculation, all NPR1 homologues were systematically identified from *Triticum aestivum* by a genome‐wide search, protein sequences from *Arabidopsis* and rice NPR family genes as query. In total, 16 NPR1‐type genes were identified in the wheat genome and were designated as TaNH1 to TaNH5 based on their relationship with the respective genes in rice (Figure S14a). Phylogenetic analysis indicated that all NPR1‐type proteins from wheat, rice and *Arabidopsis* cluster in three major groups (Figure S14a). TaNH1 was located in the same group with *Arabidopsis* NPR1. TaNH2 and TaNH3 showed high similarity with AtNPR3 and AtNPR4. Additionally, TaNH4 and TaNH5 were similar to *Arabidopsis* NPR5 and NPR6 (BOP1 and BOP2), which are mainly involved in regulating plant development (Canet *et al*., 2010). Sequence analysis showed that TaNH1, TaNH2 and TaNH3 contained an ankyrin repeat (AKR) domain, a broad complex, tramtrack, and bric‐à‐brac/poxvirus and zinc‐finger (BTB/POZ) domain and NPR1‐like domains (Figure S14b). It has been reported that the AKR domain of NPR1 is required for interacting with CIPK11 in *Arabidopsis* (Xie *et al*., 2010). Thus, we investigated the interaction between the AKR domain of TaNH1, TaNH2 or TaNH3 and TaCIPK10 by the Y2H system. Interestingly, TaCIPK10 interacted only with the AKR domain of TaNH2 (Figure S14a,c). Furthermore, the full‐length of TaNH2 showed a strong interaction with TaCIPK10. The TaNH2 mutant version, lacking the AKR domain and designated as TaNH2‐ΔAKR, failed to interact with TaCIPK10 (Figure 6a). As shown in Figure 6b, TaCIPK10 is pulled down by full‐length of TaNH2 and TaNH2‐AKR (AKR domain of TaNH2), but not by TaNH2‐ΔAKR. In addition, *in planta* interaction of TaCIPK10 with TaNH2 was tested by co‐immunoprecipitation (Co‐IP) using tobacco (*Nicotiana benthamiana*) cells transiently co‐expressing TaCIPK10 fused with Flag tag and TaNH2 or its derivatives fused with GFP. As expected, TaNH2 and TaNH2‐AKR co‐immunoprecipitated with TaCIPK10, whereas no co‐IP was detected with TaNH2‐ΔAKR (Figure 6c). Our data confirmed that TaCIPK10 interacts with TaNH2, and that the AKR motif is required for their interaction. In addition, the results of *in vitro* phosphorylation assays showed that TaCIPK10 phosphorylates TaNH2 (Figure 6d).

### TaNH2 plays a positive role in wheat resistance against *Pst*

To characterize the function of *TaNH2* in wheat resistance, the transcript accumulation of TaNH2 following biotic stress and SA treatment was investigated. As shown in Figure S10a, transcript abundance of *TaNH2* was significantly induced by *Pst,* peaking at 12 hpi during the incompatible and compatible interactions (Figure S16a). In addition, the expression level of *TaNH2* in the incompatible interaction was higher than that in the compatible interaction. Meanwhile, SA treatment elicited the significant induction of *TaNH2* as early as 2 hpt (Figure S16b).

**Figure 7:**
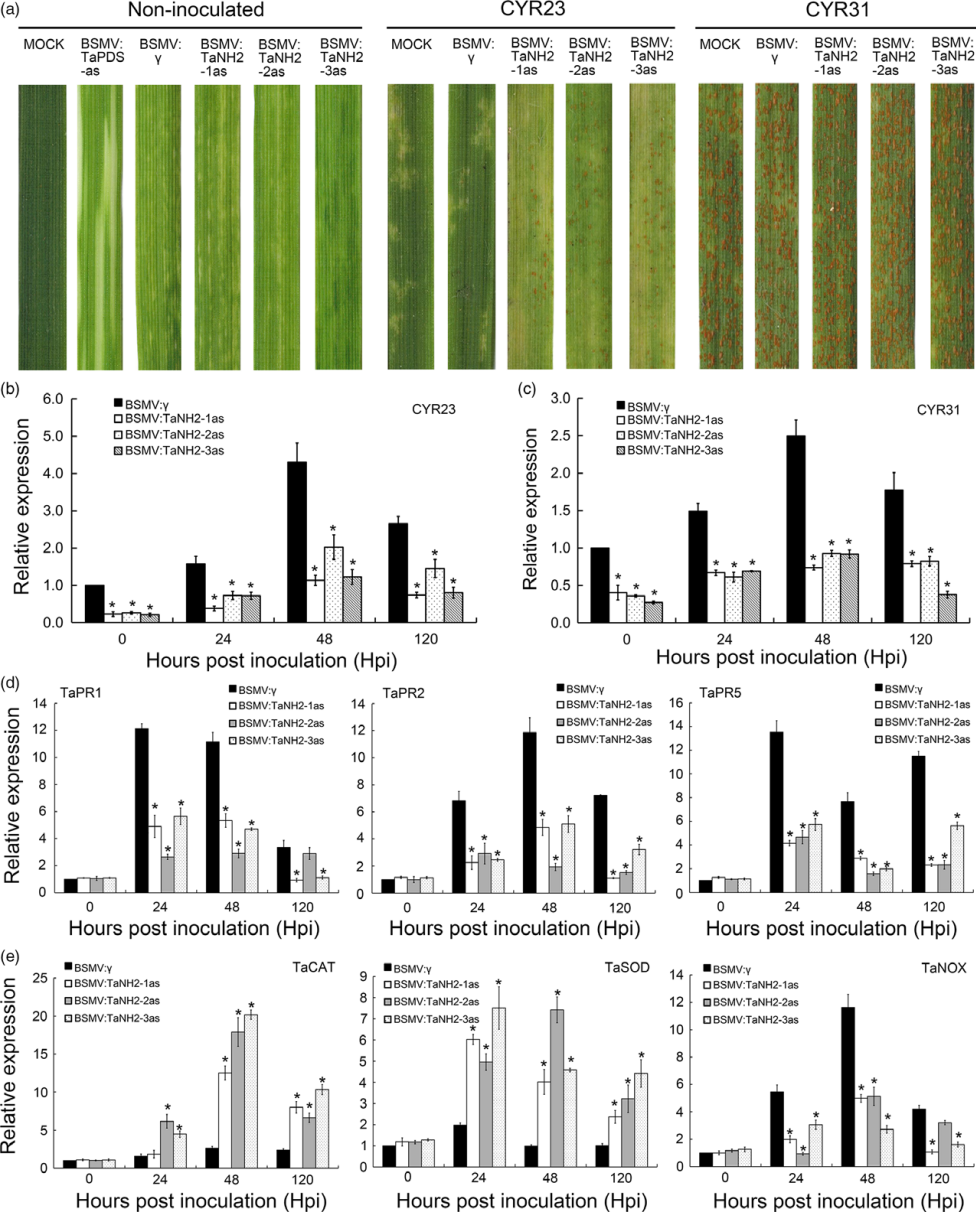
*TaNH2* positively regulates wheat resistance to *Pst*. (a) Wheat leaves of *TaNH2*‐knockdown plants were inoculated with *Pst* isolates CYR23 and CYR31 and photographed at 14 dpi. (b, c) Relative expression of *TaNH2* was decreased in *TaNH2*‐knockdown plants inoculated with CYR23 and CYR31. CK indicates BSMV:γ.The quantitative RT‐PCR values were normalized to those for *TaEF‐1α*, and are presented as fold changes relative to that in plants with BSMV:γ treatment at each time point. The transcript level of *TaCIPK10* after *Pst* inoculation in the wheat leaves with BSMV:γ treatment at each time points was standardized as 1. (d, e) Relative transcript levels of *TaPR1*,* TaPR2*,* TaPR5*,* TaSOD*,* TaCAT* and *TaNOX* were measured by qRT‐PCR during the interaction between *TaNH2*‐knockdown plants and CYR23. The quantitative RT‐PCR values were normalized to those for *TaEF‐1α*, and are presented as fold changes relative that in plants with BSMV:γ treatment at time 0. The transcript level of genes in control plants at time 0 was standardized as 1. All results were calculated by the comparative threshold (2^−ΔΔCT^) method. All data represent the mean of three biological replicates ±SE. Asterisks indicate significant differences between that in *TaNH2*‐knockdown plants and control plants using Student's *t*‐test (*P* < 0.01).

To further confirm the function of *TaNH2* during the interaction between wheat and *Pst*,* TaNH2* was knocked down by VIGS. Three fragments, showing high conservation among all copies of TaNH2 in the wheat genome database, were cloned and inserted into the virus plasmid, designated as BSMV:TaNH2‐1as, BSMV:TaNH2‐2as and BSMV:TaNH2‐3as, respectively (Figure S17). After BSMV‐inoculated plants displayed mild chlorotic mosaic symptoms at 10 dpi, the fourth leaf was inoculated with fresh urediospores of *Pst* race CYR23 and CYR31. By 14 dpi, large numbers of *Pst* uredia were produced around the necrotic spots on the leaves infected with BSMV:TaNH1/2/3as (Figure 7a). By contrast, all leaves inoculated with CYR31 produced numerous uredia (Figure 7a). The reduction in the *TaNH2* transcript level was more than 50% at each time point after CYR23 and CYR31 infection (Figure 7b,c). The expression of *TaPR1*,* TaPR2* and *TaPR5* were all reduced significantly at 24–120 hpi in *TaNH2*‐knockdown plants when challenged with the avirulent race CYR23 (incompatible interaction; Figure 7d). In addition, *TaCAT* and *TaSOD* were significantly induced, while *TaNOX* showed the opposite expression pattern in *TaNH2*‐knockdown plants against *Pst*. The histological changes associated with enhanced susceptibility of *TaNH2‐*knockdown plants to *Pst* were observed (Figure S18). The areas of cell death and H_2_O_2_ accumulation were significantly lower at 120 hpi than were observed in BSMV:γ‐treated leaves (Figure S18a,b). Additionally, the numbers of haustoria and haustorial mother cells, the hyphal lengths and infection area were significantly increased in all *TaNH2*‐knockdown plants inoculated with CYR23 (Figure S18c–f). Therefore, knocking down *TaNH2* reduces wheat resistance to *Pst*.

## Discussion

As a second messenger, Ca^2+^ undergoes transient changes in concentrations in response to various stimuli and regulates numerous inter‐ and extra‐cellular signalling processes (Ghosh and Greenberg, 1995). However, the function of Ca^2+^‐regulated signals in immune signalling of crop plants is largely unknown. In this study, we identified and characterized a SA‐induced *CIPK,* TaCIPK10, which is regulated by Ca^2+^ sensor proteins, TaCBLs. A series of complementary cellular‐ and genetics‐based assays demonstrated that TaCIPK10 performs a crucial role in wheat resistance to *Pst* and TaNH2 functions as the interact partner of TaCIPK10 to regulate wheat defense response. Our results suggested that Ca^2+^‐regulated signal play an important role in wheat resistance and NPR1‐like protein is involved in Ca^2+^‐regulated signal.

### TaCIPK10 positively regulate wheat resistance to *Pst* as the downstream of Ca^2+^ signal

In *Arabidopsis* and rice, the protein kinase activity of CIPKs have been shown to depend on CBLs and external Ca^2+^ (Halfter *et al*., 2000; Kurusu *et al*., 2010). Similarly, the kinase activity of TaCIPK10 is regulated by TaCBL4 and Ca^2+^. These results suggest that the regulation of CIPKs protein kinase activity is very conservative among all plants. Moreover, the influx of Ca^2+^ from the intercellular space to the cytoplasm was promoted as earlier as 12 hpi during the incompatible interaction between wheat and *Pst* (Yin *et al*., 2015). *TaCIPK10* expression was peaked at 12 hpi in the incompatible interaction (Figure 1b). All these results suggested that TaCIPK10 may be activated by *Pst* infection through TaCBLs binding to Ca^2+^ and inactivated at a resting cytosolic Ca^2+^ level. In addition, TaCIPK10 overexpression enhanced wheat resistance to *Pst* for the induction of a wide range of defense response, including hypersensitive cell death, ROS generation and PR gene expression. On the other hand, *TaCIPK10*‐knockdown plants significantly reduced wheat resistance to *Pst* (Figures 4 and 5). Our results confirmed that TaCIPK10 play a positive role in wheat resistance. In *Arabidopsis*,* cipk23* mutant exhibited severe sensitivity to low‐K conditions (Xu *et al*., 2006). Furthermore, AtCIPK23 was identified to be involved in Ca^2+^‐dependent regulation of low‐K^+^ tolerance and adaptation by detailed biochemical, electrophysiological and genetic‐based approaches (Li *et al*., 2006). Thus, our results suggested that TaCIPK10 positively regulate wheat resistance to *Pst* as molecular links between of Ca^2+^ and downstream components of defense response.

### The function of TaCIPK10 in wheat resistance to stripe rust may depend on SA signal

Salicylic acid has confirmed to be synthesized by plants in response to challenge by a diverse range of pathogens (Vlot *et al*., 2009). Similarly, SA was induced by *Pst* infection in wheat, which suggested that SA is involved in wheat resistance to *Pst* (Figure S2). With induction of TaCIPK10 expression under SA treatment, we suggested that TaCIPK10 is involved in SA signal. During the interaction between TaCIPK10 overexpression transgenic lines and *Pst*, the PR genes were significantly induced by *Pst* infection compared to control (Figure 5), while exhibiting the opposite expression pattern in TaCIPK10‐knockdown plants. PRs have been shown to act as disease resistance factors and can be induced by SA (Loon *et al*., 2006). Particularly, PR1 is the marker gene in SA‐dependent responses (Fu and Dong, 2013). Moreover, AtNPR3 and AtNPR4 have been shown to regulate SA‐dependent immunity in *Arabidopsis* (Fu *et al*., 2012). Among all identified NPR1 homologue proteins in wheat, TaNH2, which shared high similarity with AtNPR3/4, was confirmed to interact with and be phosphorylated by TaCIPK10. Our results suggested that the function of TaCIPK10 in wheat resistance to stripe rust may depend on SA signal.

In rice, OsCIPK14/15 was confirmed to regulate MAMP (Microbe‐Associated Molecular Pattern) induced hypersensitive cell death, phytoalexin production and defense gene expression (Kurusu *et al*., 2010). Moreover, tomato CBL10‐CIPK6 complex contributes to ROS generation during effector‐triggered immunity in the interaction of *Pseudomonas syringae* pv tomato DC3000 (Drerup *et al*., 2013). Another CBL‐CIPK complex in wheat, TaCB4‐TaCIPK5, has been confirmed to positively contribute to wheat resistance to *Pst* in ROS‐depend manner (Liu *et al*., 2018). Together with our result in this study, we suggested that CIPKs in plant kingdom not only participate in abiotic stress, but also play a crucial role in plant response to biotic stress.

### TaNH2 may be involved in Ca^2+^‐regulated signal during the interaction between wheat and *Pst*

The structure of proteins is regulated by their phosphorylation, normally accompanied by modulating protein activity and altered interaction partners or subcellular localization (Schulze, 2010). In *Arabidopsis*, Ser‐589 of AtNPR1 being phosphorylated by NF1‐related kinase 2.8 (SnRK2.8) was found to be required for nuclear import of AtNPR1 during SAR (Lee *et al*., 2015), indicating that AtNPR1 is involved in SnRK2.8‐mediated plant immunity. In our study, TaCIPK10 phosphorylated TaNH2 and both TaNH2 and TaCIPK10 play a positive role in wheat resistance to *Pst*. Thus, we suggested that TaNH2 functions as the interact partner of TaCIPK10 to regulate wheat resistance to *Pst*. In *Arabidopsis*, SOS1, a Na^+^/H^+^ antiporter, phosphorylated and activated by SOS2/CIPK24‐SOS3/CBL4 complex at plasma membrane, indicating that SOS1 plays very important role in transducing Ca^2+^ signal for providing salt tolerance (Shi *et al*., 2000; Zhu, 2002). Moreover, AtCIPK26 after interacting with the calcium sensors AtCBL1 or AtCBL9 enhances the activity of the NADPH oxidase RBOHF via phosphorylation, demonstrating AtRBOHF participated in Ca^2+^ induced ROS generation (Drerup *et al*., 2013). As TaCIPK10 was regulated by Ca^2+^, we suggested that TaNH2 is involved in Ca^2+^‐regulated signal during the interaction between wheat and *Pst*.

### The mechanism of NPR1 homologues mediated disease resistance in wheat is different to that in *Arabidopsis*

In our study, the expression of *TaNH2* was found to be induced by *Pst* infection. As a previous study shown (Wang *et al*., 2010), the up‐regulation of host genes during infection may indicate the exploitation of cellular resources and/or the activation of defense responses, suggesting that TaNH2 is involved in wheat resistance. In addition, TaNH2 was shown to positively regulate wheat resistance characterized by decreased necrotic area, reduced ROS accumulation and increased uredia production in *TaNH2*‐knockdown plants (Figure 7 and Figure S15). Similarly, OsNH3/OsNPR3, which located in the same group with TaNH2, has been confirmed to play a positive role in rice disease resistance to bacterial blight (Bai *et al*., 2011). However, AtNPR3/4, which shared higher similarity with TaNH2, has been shown to play a negative role in plant immunity (Fu *et al*., 2012). Additionally, The NPR1 paralogues NPR3 and NPR4 are involved in the degradation of NPR1 in a SA concentration‐dependent manner, which is required for SAR establishment in *Arabidopsis* (Fu *et al*., 2012). In wheat, initial pathogen infection can induce plant resistance to secondary pathogen infection, but not systemic (Wang *et al*., 2018). These different biological processes caused by NPR1 homologue genes in wheat and *Arabidopsis* seem to be achieved by activating different signal pathways. All these different results indicate that the mechanism of NPR1 homologues mediated disease resistance in cereal plants is different to that in *Arabidopsis*.

To our knowledge, this is the first direct evidence demonstrating that the NPR1 homologue gene is involved in Ca^2+^‐regulated signal during the interaction between wheat and *Pst* (Figure S19). Additionally, the overexpression transgenic wheat, which was used for investigating the function of TaCIPK10 in wheat resistance, has potential as an alternative material in conventional breeding for creating environmentally friendly and durable resistant varieties. In conclusion, the concentration of Ca^2+^ in plant cells is affected by *Pst* infection to secure the kinase activity of TaCIPK10, and TaNH2 functions as a phosphorylation substrate of TaCIPK10 for transmitting Ca^2+^ signals that are important in conferring wheat resistance. We suggest that TaNH2 participate in Ca^2+^‐regulated signal in wheat defense response. Indeed, our findings support the notion that *NPR1*‐like genes and the genes involved in CBL‐CIPK pathway co‐regulate wheat resistance against *Pst*.

## Experimental procedures

### Plant material, fungal pathogens and treatments

Wheat (*T. aestivum* L.) genotype Suwon 11 and isolates of *Pst* CYR23 (avirulent) or CYR31 (virulent) were used in this study. Suwon 11, carrying the *YrSu* resistance gene, is resistant to CYR23 infection, but is highly susceptible to CYR31 (Cao *et al*., 2002). The second leaves inoculated with CYR23, CYR31 or sterile distilled water (control) were harvested at 0, 6, 12, 18, 24, 48, 72 and 120 h postinoculation (hpi) for RNA extraction. Time points were selected based on a previous study (Wang *et al*., 2007). For biological stress treatments, wheat seedlings were grown, inoculated and maintained as described previously (Tang *et al*., 2015). For chemical treatments, 10‐day‐old wheat seedlings were sprayed with 2 mm salicylic acid (SA) dissolved in 0.1% (v/v) ethanol. Mock control plants were sprayed with 0.1% ethanol. Samples were collected at 0, 4, 6, 8, 12 and 24 h posttreatment (hpt) for RNA extraction and qRT‐PCR. XN1376, an early maturing winter wheat variety, was used for wheat transformation and expressed high susceptibility to *Pst* race CYR32. Methods of gene cloning, sequence analysis DNA and RNA extraction, cDNA synthesis and quantitative real‐time PCR were performed following a previous study (Liu *et al*., 2018). To measure fungal biomass, the relative quantification of the wheat gene *TaEF‐1a* and the *Pst* gene *PstEF1* (Yin *et al*., 2009) was assessed. The relative quantities of the PCR product of *PstEF1* and *TaEF‐1α* in infected samples were calculated using the gene‐specific standard curves to quantify the *Pst* and wheat DNA, respectively.

### Yeast two‐hybrid assays

For interaction analysis of TaCIPK10 and TaCBLs or TaNH2, the MatchMaker yeast two‐hybrid system was performed (Takara, Dalian, China). The coding sequences of *TaCBLs*,* TaNH2*,* TaNH2‐ΔAKR* and the AKR motif of TaNH1/2/3 were subcloned into pGADT7 (activation domain, AD), the coding sequences of *TaCIPK10* and *TaCIPK10Δ* were subcloned into pGBKT7 (DNA‐binding domain, BD) vector. The pairs of recombinant plasmids of AD and BD were co‐transformed into yeast strain AH109 following the Yeast Protocols Handbook (Takara, Dalian, China). Protein expression in yeast was confirmed by Western blots following a previous study (Wang *et al*., 2016).

### Site‐directed mutagenesis and deletion mutants

For site mutagenesis and creation of deletion mutants, the overlap‐PCR approach was used. First‐round PCR products were amplified with the following oligonucleotides: TaCIPK10(K)‐S/AS for TaCIPK10(K42N), TaCIPK10(S)‐S/AS for TaCIPK10(S157D), TaCIPK10(T)‐S/AS for TaCIPK10(T171D), TaCIPK10(Y)‐S/AS for TaCIPK10(Y178D), TaCIPK10(M)‐S/AS for TaCIPK10(ΔNAF) (Table S2). The PCR products were used as templates for amplification with TaCIPK10‐32a‐S/AS. Each final PCR fragment was cloned into pGEM‐T Easy Vector (Promega, Madison, WI) for sequencing.

### Preparation and purification of recombinant proteins

The prokaryotic expression constructs were created by cloning the PCR‐amplified ORF of wheat CIPK10, NH2 or their mutants into *Bam*HI and *Eco*RI sites of pET‐32a or pGEX‐4t‐1 expression vector containing His‐Tag or GST‐Tag sequence. *Escherichia coli* BL21 (DE3) strain cells transformed with the resulting vectors were grown in 10‐mL Luria‐Bertani (LB) medium at 37 °C overnight, and the fresh cultures of *E. coli* carrying the foreign‐genes were subcultured until the A600 reached 0.8 in 200‐mL LB. His‐tag fusion proteins were induced by adding 0.5 m Isopropyl β‐D‐Thiogalactoside (IPTG) at 25 °C overnight. The cells were harvested by centrifugation, resuspended in cold lysis buffer [100 mm Tris (pH 7.5), 150 mm NaCl, and 10 mm Imidazole] and lysed by ultrasonic fragmentation in accordance with the supplier's instructions of ultrasonic instrument (SCIENTZ, Ningbo, China; Amplitude 40%, Pulse on 4 s, Pulse off 6 s, total time 15 min). The cell lysate was centrifuged at 20 000 ***g*** for 30 min at 4 °C. The supernatant was applied onto a Ni‐NTA agarose (QIAGEN, Düsseldorf, Germany) according to the manufacturer's instructions. Purified proteins were eluted with 1‐mL of wash buffer (100 mm Tris (pH 7.5), 150 mm NaCl, and 200 mm Imidazole) and used for the kinase activity assay.

### Protein kinase assay

To assay autophosphorylation activity, purified TaCIPK10 and mutant version proteins were incubated in kinase buffer [10 μCi of (γ‐32P) ATP, 20 mm Tris‐HCl pH 7.5, 1 mm DTT, 20 mm MgCl_2_, 50 μm ATP and either 5 mm CaCl_2_ or ETDA] for 60 min at 37 °C in a final volume of 25 μL. The reaction was stopped by addition of 5 μL of 4 × Laemmli loading buffer, boiled immediately for 5 min, and analysed on 12% SDS‐PAGE gels. Gels were stained with Coomassie Brilliant Blue, dried and visualized by autoradiography. TaCIPK10 kinase transactivity was determined in the presence of 100 ng of His‐TaCBL4 or His‐TaCBL6 fusion protein and 3 μg of MBP (Roche) were added to the kinase reaction mixture and incubated at 30 °C for 60 min. Kinase activity was visualized by autoradiography. For phosphorylation of TaNH2 or its mutants, the reactions were initiated by mixing 500 ng of TaCIPK10 or TaCIPK10‐K42N and 1 μg TaNH2 or its mutants in 25 μL kinase buffer in the presence of 100 ng of His‐TaCBL4. The details of preparation and purification of recombinant proteins are described in the [Link], [Link].

### Virus‐induced gene silencing

Three specific cDNA fragments of *TaCIPK10* or *TaNH2* with *Not*I and *Pac*I restriction sites were obtained by reverse transcription PCR for VIGS analysis as previously described (Holzberg *et al*., 2002). In addition, we confirmed that the fragments showed no similarity with any other genes by BLAST analysis of the wheat genome databases (https://urgi.versailles.inra.fr/blast/). The capped *in vitro* transcripts of BSMV RNA were prepared from linearized plasmids containing the tripartite BSMV genome using the Message T7 *in vitro* transcription kit (Ambion, Austin, TX; Promega, Shenzhen, China) following the manufacturer's instructions. The more details of VIGS assay, host response detection and the methods of histological observation for fungal growth were performed according to a previous study (Liu *et al*., 2018).

### Plant transformation

The *TaCIPK10* ORF sequence was subcloned into the *Sma*I site of the modified expression vector pWMB003, resulting in the transformation vector pWMB003‐TaCIPK10. The herbicide tolerance gene, *Bar*, was used as a selectable marker gene in vector pAHC20. The vector pAHC20 and pWMB003‐TaCIPK10 were co‐transformed into wheat calli by particle bombardment. The methods of regeneration and selection were carried out according to a previous study (Qi *et al*., 2017). The second leaf of T_2_ generation wheat was inoculated with urediospores of CYR32. These leaves were collected at 0, 24, 48 and 120 hpi for RNA isolation and histological observation.

### GST pull down and Co‐immunoprecipitation (Co‐IP) assay

The coding sequences of *TaNH2*,* TaNH2‐AKR* and *TaNH2‐ΔAKR* were subcloned into *Bam*HI and *Eco*RI sites of pGEX‐4T‐1 expression vector containing the GST‐Tag sequence. GST fusion proteins of TaNH2 or derivatives were expressed in *E. coli* (BL21) by induction with 0.5 mm IPTG at 25 °C overnight and purified by standard techniques with glutathione‐sepharose following the previous study (Harper and Speicher, 2001). Equal amounts of GST‐tagged proteins were mixed with TaCIPK10‐His and incubated at 4 °C for 2 h followed by washing and protein blots to detect the recovered TaCIPK10‐His levels using the GST Protein Interaction Pull‐Down Kit (Thermos, Shanghai, China) according to the manufacturer's instructions. Anti‐His (Sungene, Tianjing, China) at 1:5000 dilution and anti‐GST (Sungene) at 1:5000 dilution were used for the Western blots. The way of Co‐ip assay were performed as described in a previous study (Liu *et al*., 2018).

### Statistical analysis

Microsoft Excel software was used to calculate mean values and standard errors. Student's *t*‐test performed by the SPSS 16.0 (SPSS Inc., Chicago, IL) statistical software was used to determine the significant differences between control and treatment or between time‐course points. The significant change with unequal variance was measured by probability (*P*) value <0.01.

### Accession numbers

The nucleotide sequences used in this study had the following GenBank accession numbers: *TaEF‐1α* (elongation factor‐1, Q03033), *TaCBL1.1* (KU736847), *TaCBL2* (KU736848.1), *TaCBL3* (KU736849), *TaCBL4* (KU736850.1), *TaCBL6* (KU736851.1), *TaCBL9* (KU736852.1), *TaCIPK10* (KU736856.1), *TaNH2* (KU736862.1), *TaPR1* (pathogenesis‐related protein 1, AF384143), *TaPR2* (*β*‐1,3‐glucanase, DQ090946), *TaPR5* (thaumatin‐like protein; FG618781), *TaSOD* (superoxide dismutase, CB307850), *TaCAT* (catalase, X94352) and *TaNOX* (AY561153).

## Funding information

National Key R&D Program of China (2018YFD0200400), Natural Science Basic Research Plan in Shaanxi Province of China (2017JM3007) and National Natural Science Foundation of China (31620103913).

## Author contributions

Z. K., J. G. (Jun Guo), P. L., J. G. (Jia Guo) and Y. D. designed experiments. P. L., R. Z., J. Z., C. L., T. Q. and Y. D. and performed the experiments. J. G. (Jia Guo) did the phylogenetic analysis. P. L and J. G. (Jun Guo) wrote the manuscript.

## Conflicts of interest

The authors declare no conflicts of interest.

## Supporting information


**Figure S1** Transcript profiles of selected *TaCIPKs* in wheat leaves in response to SA treatment and *Pst* inoculation.
**Figure S2** Infection of *Pst* race CYR23 (incompatible interaction) or CYR31 (compatible interaction) differentially increased the endogenous level of SA in wheat leaves of cultivar Suwon11.
**Figure S3** Multiple sequence alignments of the coding sequences for the three *TaCIPK10* copies.
**Figure S4** TaCIPK10 protein structure and phylogenetic analysis.
**Figure S5** Subcellular localization of TaCIPK10 in wheat protoplasts.
**Figure S6** Western blot analysis of protein expression in yeast two‐hybrid assays.
**Figure S7** Multiple sequence alignment of *TaCIPK10* and three *TaCIPK* members.
**Figure S8** Relative transcript levels of *TaPR1*,* TaPR2*,* TaPR5*,* TaSOD*,* TaCAT* and *TaNOX* in *TaCIPK10*‐knockdown plants inoculated with avirulent race, CYR23.
**Figure S9** Host response in *TaCIPK10*‐knockdown plants inoculated with *Pst* avirulent race CYR23.
**Figure S10** Growth of *Pst* avirulent race, CYR23, was increased in *TaCIPK10*‐knockdown plants.
**Figure S11** Functional analysis of *TaCIPK10* overexpression transgenic wheat.
**Figure S12** Histological observations of positive *TaCIPK10* overexpression lines infected with virulent race CYR32 at 120 hpi.
**Figure S13** TaCIPK10 overexpression transgenic lines did not affect the growth of wheat.
**Figure S14** TaCIPK10 interacted only with AKR motif of TaNH2.
**Figure S15** Schematic diagrams of TaNH2.
**Figure S16** *TaNH2* was significantly induced by SA treatment and *Pst* inoculation.
**Figure S17** Sequence alignment of the coding sequences for the three *TaNH2* copies in the wheat genome database and TaNH2 cloned from Suwon 11.
**Figure S18** Host response and histological observations of fungal growth in *TaNH2*‐knockdown plants challenged by avirulent CYR23.
**Figure S19** Schematic presentation of possible molecular mechanism of *TaCIPK10*‐mediated resistance processes relative to NPR1‐like gene in wheat.

**Table S1** Disease severity scale.
**Table S2** Primers used in this study.
