# Supplementary material for: TaCIPK10 interacts with and phosphorylates TaNH2 to activate wheat defense responses to stripe rust
Source: Plant Biotechnol J. 2018 Dec 5;17(5):956–68. doi: 10.1111/pbi.13031 (PMC6587807; doi:10.1111/pbi.13031)
Supplement: Supplementary file 1 — Figure S1 Transcript profiles of selected TaCIPKs in wheat leaves in response to SA treatment and Pst inoculation. Figure S2 Infection of Pst race CYR23 (incompatible interaction) or CYR31 (compatible interaction) differentially increased the endogenous level of SA in wheat leaves of cultivar Suwon11. Figure S3 Multiple sequence alignments of the coding sequences for the three TaCIPK10 copies. Figure S4 TaCIPK10 protein structure and phylogenetic analysis. Figure S5 Subcellular localization of TaCIPK10 in wheat protoplasts. Figure S6 Western blot analysis of protein expression in yeast two‐hybrid assays. Figure S7 Multiple sequence alignment of TaCIPK10 and three TaCIPK members. Figure S8 Relative transcript levels of TaPR1, TaPR2, TaPR5, TaSOD, TaCAT and TaNOX in TaCIPK10‐knockdown plants inoculated with avirulent race, CYR23. Figure S9 Host response in TaCIPK10‐knockdown plants inoculated with Pst avirulent race CYR23. Figure S10 Growth of Pst avirulent race, CYR23, was increased in TaCIPK10‐knockdown plants. Figure S11 Functional analysis of TaCIPK10 overexpression transgenic wheat. Figure S12 Histological observations of positive TaCIPK10 overexpression lines infected with virulent race CYR32 at 120 hpi. Figure S13 TaCIPK10 overexpression transgenic lines did not affect the growth of wheat. Figure S14 TaCIPK10 interacted only with AKR motif of TaNH2. Figure S15 Schematic diagrams of TaNH2. Figure S16 TaNH2 was significantly induced by SA treatment and Pst inoculation. Figure S17 Sequence alignment of the coding sequences for the three TaNH2 copies in the wheat genome database and TaNH2 cloned from Suwon 11. Figure S18 Host response and histological observations of fungal growth in TaNH2‐knockdown plants challenged by avirulent CYR23. Figure S19 Schematic presentation of possible molecular mechanism of TaCIPK10‐mediated resistance processes relative to NPR1‐like gene in wheat. [file PBI-17-956-s002.docx]

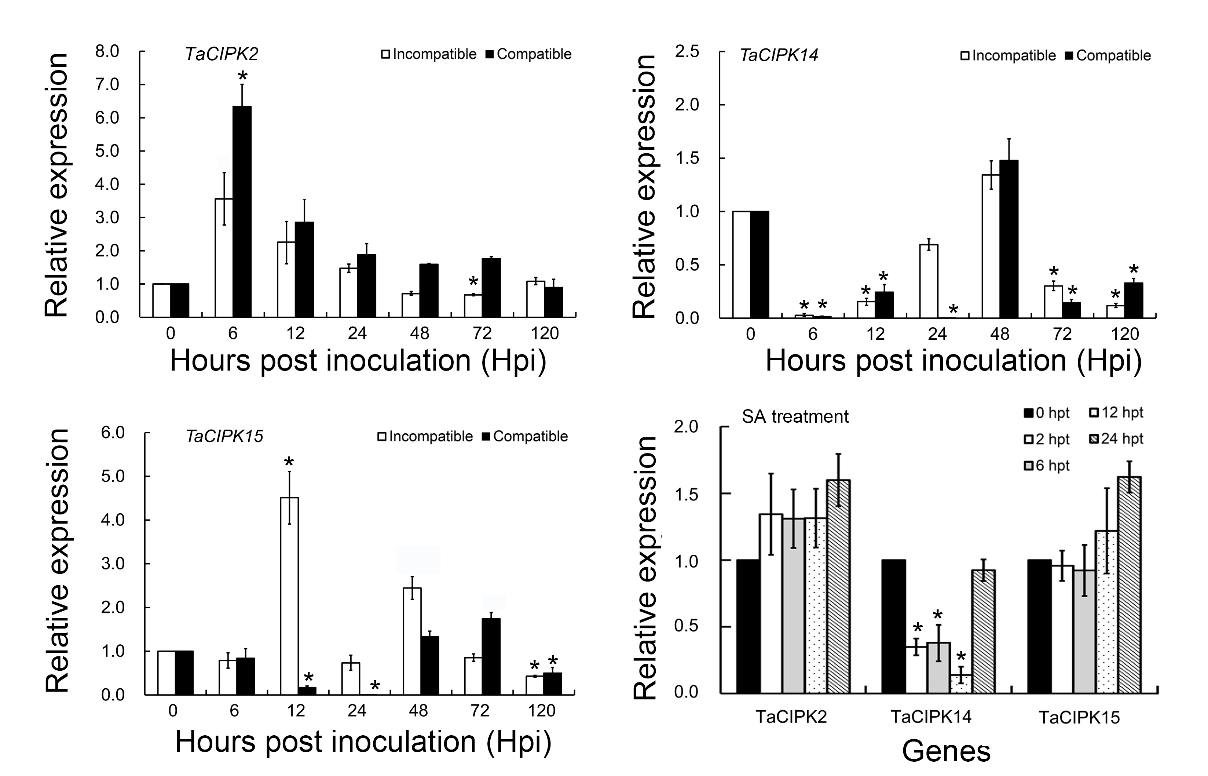


**Figure S1 Transcript profiles of selected *TaCIPKs* in wheat leaves in response to SA treatment and *Pst* inoculation.** Wheat leaves inoculated with *Pst* isolates CYR23 (incompatible interaction) and CYR31 (compatible interaction) were sampled at 0, 6, 12, 24, 48, 72, and 120 hours post inoculation (hpi) or treated with 2 mM SA and were collected at 0, 2, 6, 12 and 24 hours post treatment (hpt). Relative transcript levels of *TaCIPKs* were calculated by the comparative threshold (2^-ΔΔCT^) method. The quantitative RT-PCR values were normalized to those for *TaEF-1α* and are presented as fold changes relative to that in un-inoculated plants at time 0. The transcript level of TaCIPKs in the wheat leaves at time 0 was standardized as 1. Each data point represents mean (3 replicates) +/- standard deviation. Asterisks indicate significant differences between time-course points using Student t’ test (P < 0.01).


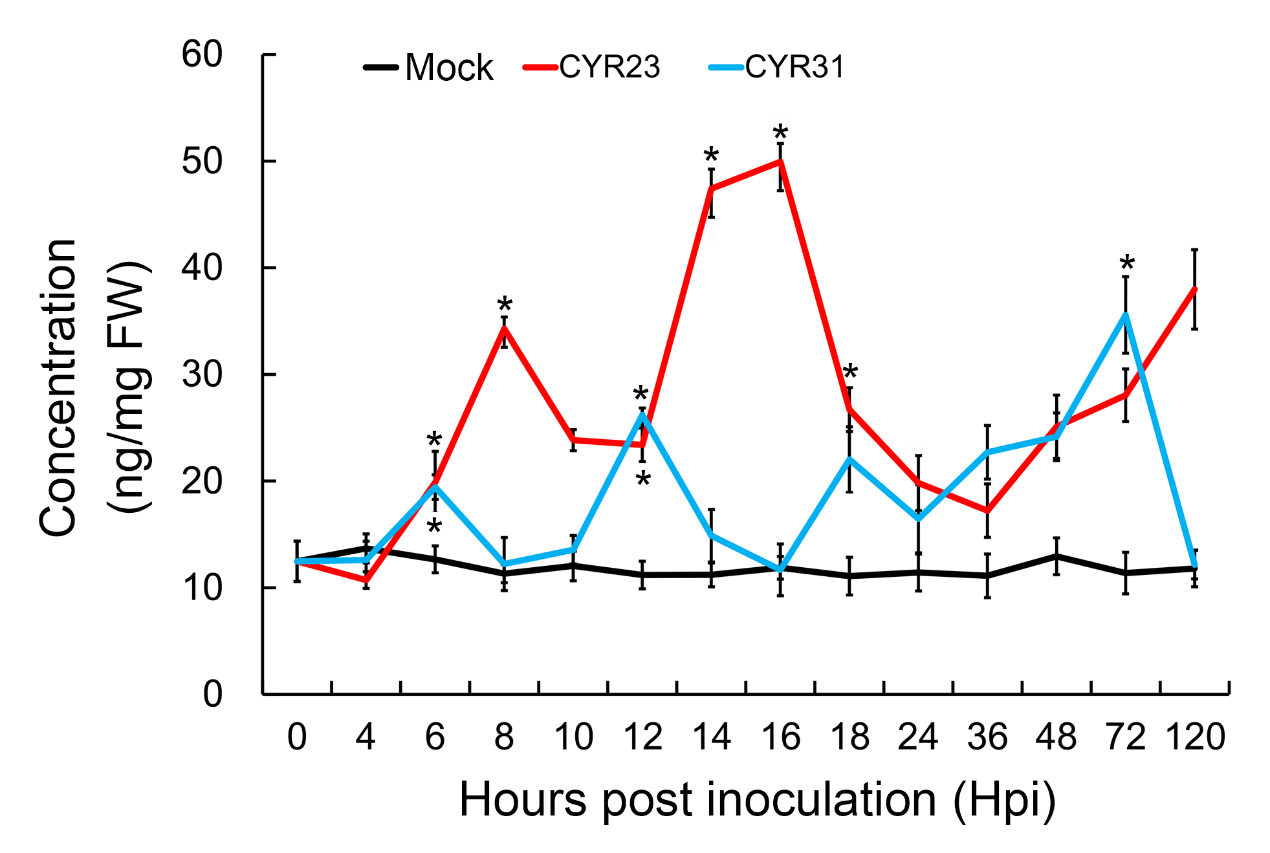


**Figure S2 Infection of *Pst* race CYR23 (incompatible interaction) or CYR31 (compatible interaction) differentially increased the endogenous level of SA in wheat leaves of cultivar Suwon11.** Wheat leaves inoculated with Pst race CYR23 and CYR31 were sampled at 0, 4, 6, 8, 10, 12, 14, 16, 18, 24, 36, 48, 72 and 120 hpi, respectively. CK, wheat leaves inoculated with water. Each data point represents mean (2 replicates) +/- standard deviation. Asterisks indicate significant differences between in wheat leaves with Pst infection and uninfected leaves at the same time points using Student t’ test (P < 0.01).


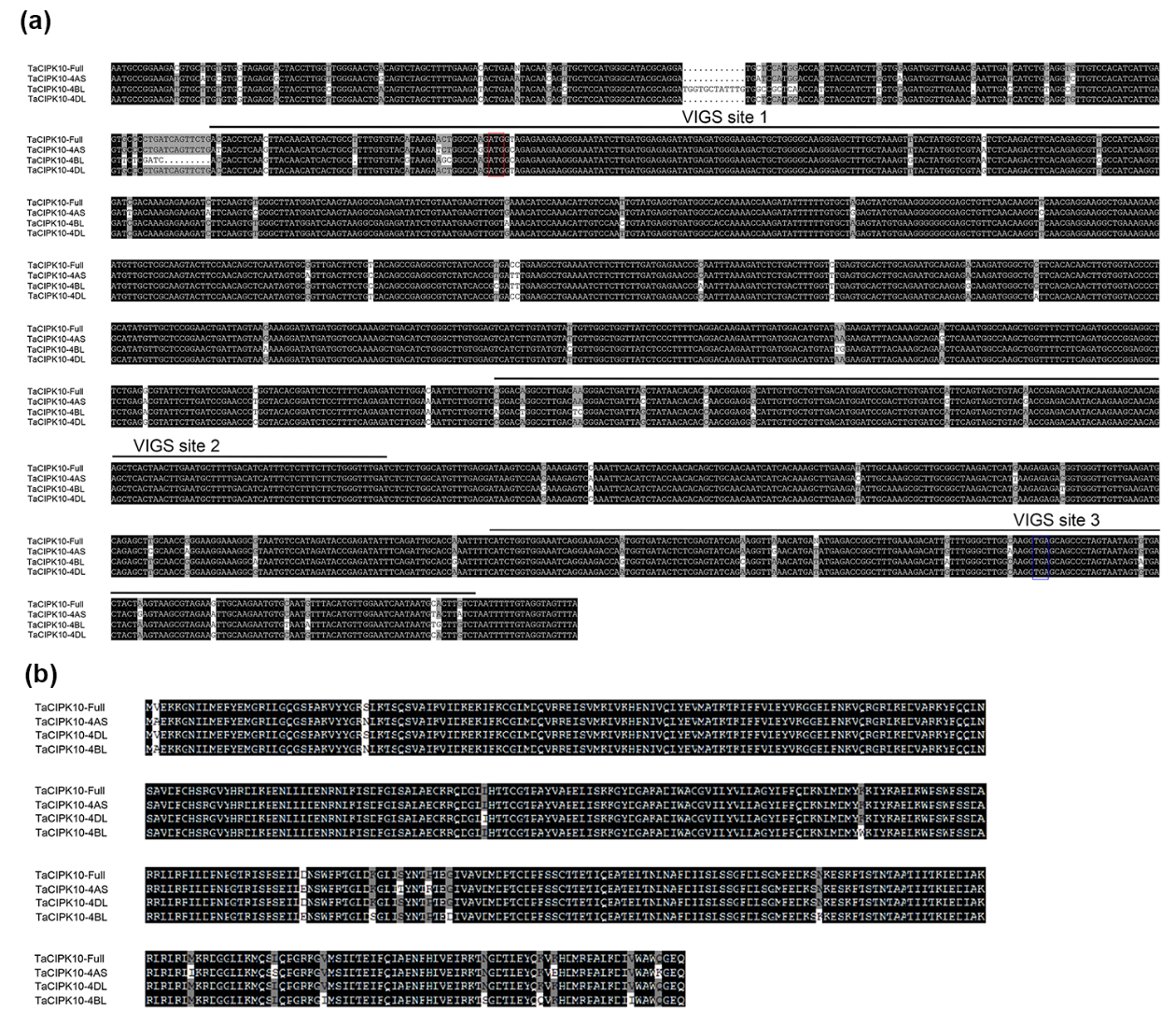


**Figure S3 Multiple sequence alignment of the coding sequences for the three *TaCIPK10* copies.** **a**, The coding sequences of three *TaCIPK10* copies in genome database of wheat cv. Chinese Spring or cloned from wheat cv. Suwon11 were aligned by DNAMAN software. Red box: initiation codon (ATG), blue box: termination codon (TGA). The fragments for VIGS are indicated by overbars. VIGS site 1 to site 3 represent for TaCIPK10-1as to TaCIPK10-3as, respectively. **b**, Multi-sequence alignment of the deduced TaCIPK10 proteins from wheat cv. Suwon11 and the three copies from Chinese Spring genome database.


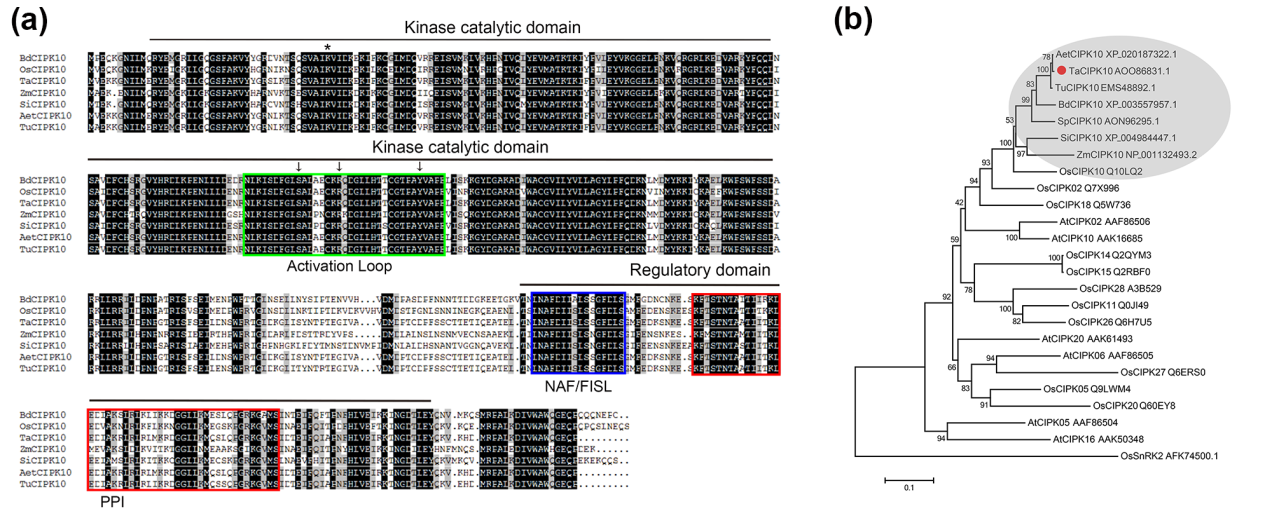


**Figure S4 TaCIPK10 protein structure and phylogenetic analysis. a**, Multiple sequence alignment of TaCIPK10 against CIPK10 from other species were performed by DNAMAN software. Identical amino acid residues in all eight sequences are highlighted in black, and similar residues are highlighted in gray. The activation loop is denoted by a green box. The conserved NAP/FISL motif is denoted by a blue box and the protein-phosphatase interaction (PPI) motif is marked by a red box. The asterisk indicates the conserved ATP-binding residue Lys and the black arrows indicate three putative trans-phosphorylation sites, Thr, Ser and Tyr. **b**, Phylogenetic analysis of CIPK10 protein sequences based on multiple alignments. The number above the internal branches indicates bootstrap values estimated based on 500 replications. Branches are labeled with protein names and GenBank accession number. Aet, *Aegilops tauschii*; Ta, *Triticum aestivum*; Tu, *Triticum urartu*; Bd, *Brachypodium distachyon*; Sp, *Stipa purpurea*; Os, *Oryza sativa*; Zm, *Zea mays*; Si, *Setaria italica*; At, *Arabidopsis thaliana*. OsSnRK2 was used as the outgroup.


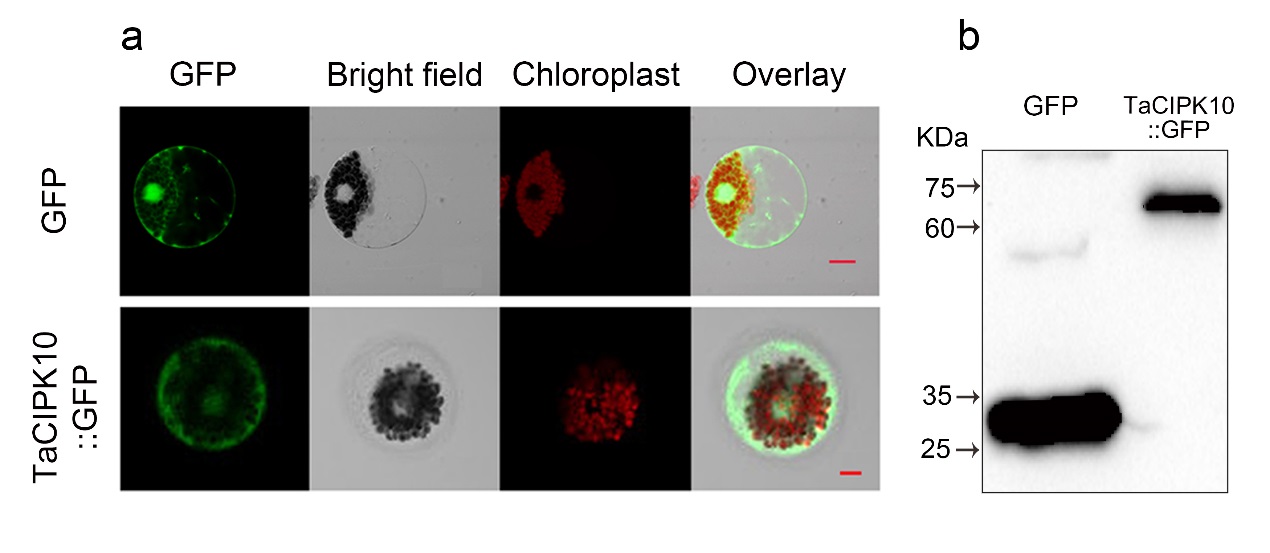


**Figure S5 Subcellular localization of TaCIPK10 in wheat protoplasts. a,** TaCIPK10 was fused to GFP and transiently expressed in wheat protoplasts. The green color indicates GFP signals. Bar, 5μm. **b**, the expression of TaCIPK10 fusion protein in wheat protoplasts was detected by western blot using anti-GFP antibody. Number near the bands indicates the marker.


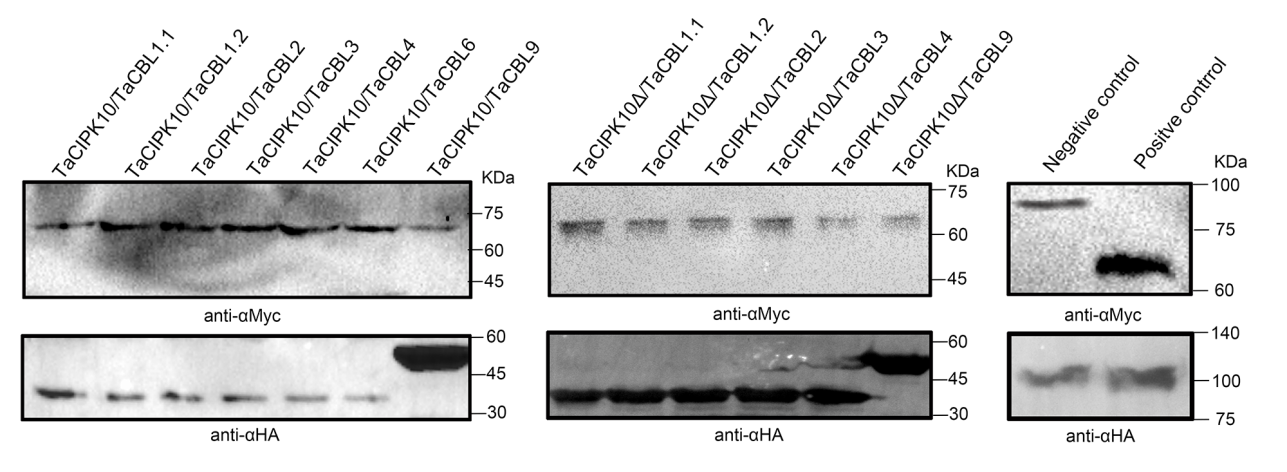


**Figure S6 Western blot analysis of protein expression in yeast two-hybrid assays.** Western blot assays were performed to validate the protein expressed by either AD or BD vectors using anti-HA and anti-αMyc antibodies.


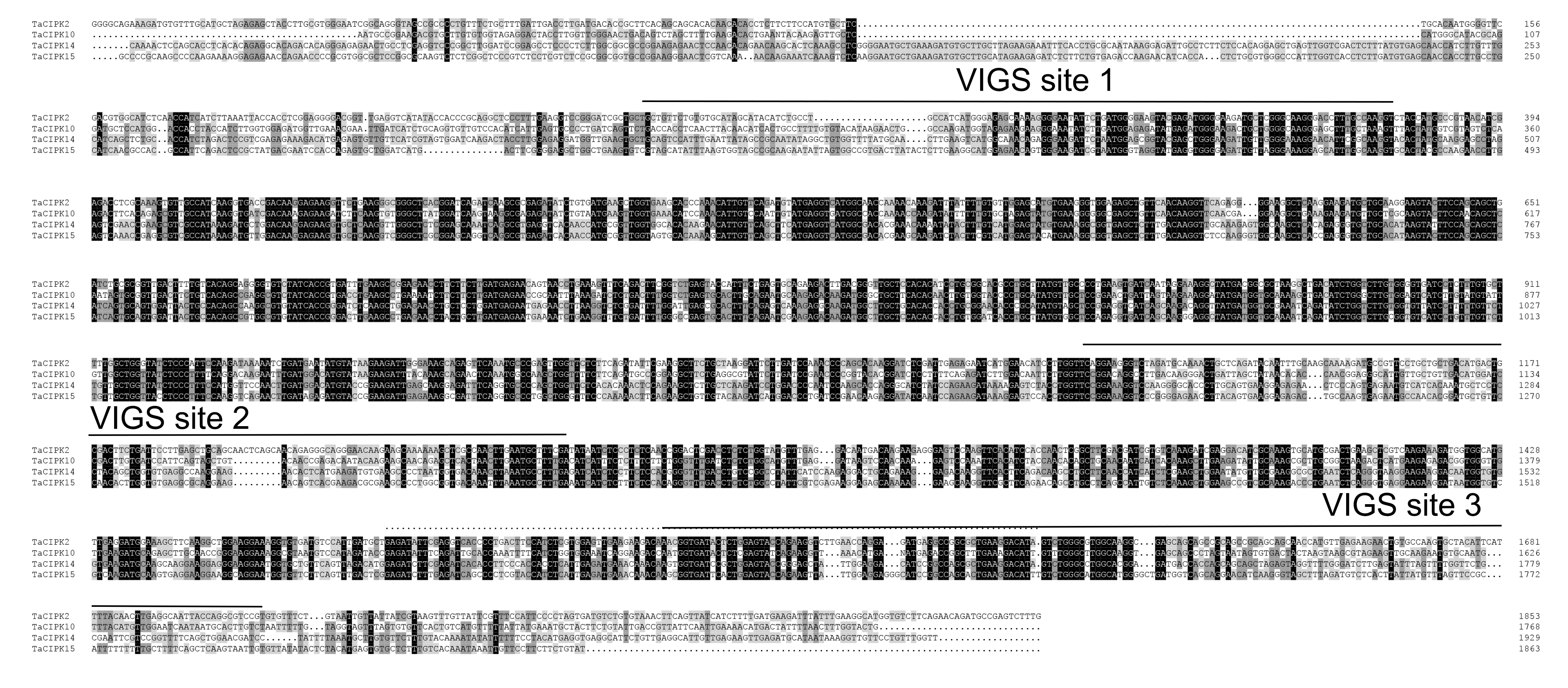


**Figure S7 Multiple sequence alignment of *TaCIPK10* and three *TaCIPK* members.** Several TaCIPKs, located within the same clade with TaCIPK10, were selected for sequence alignment. VIGS site 1-3 represent TaCIPK10-1as, TaCIPK10-2as and TaCIPK10-3as, respectively.


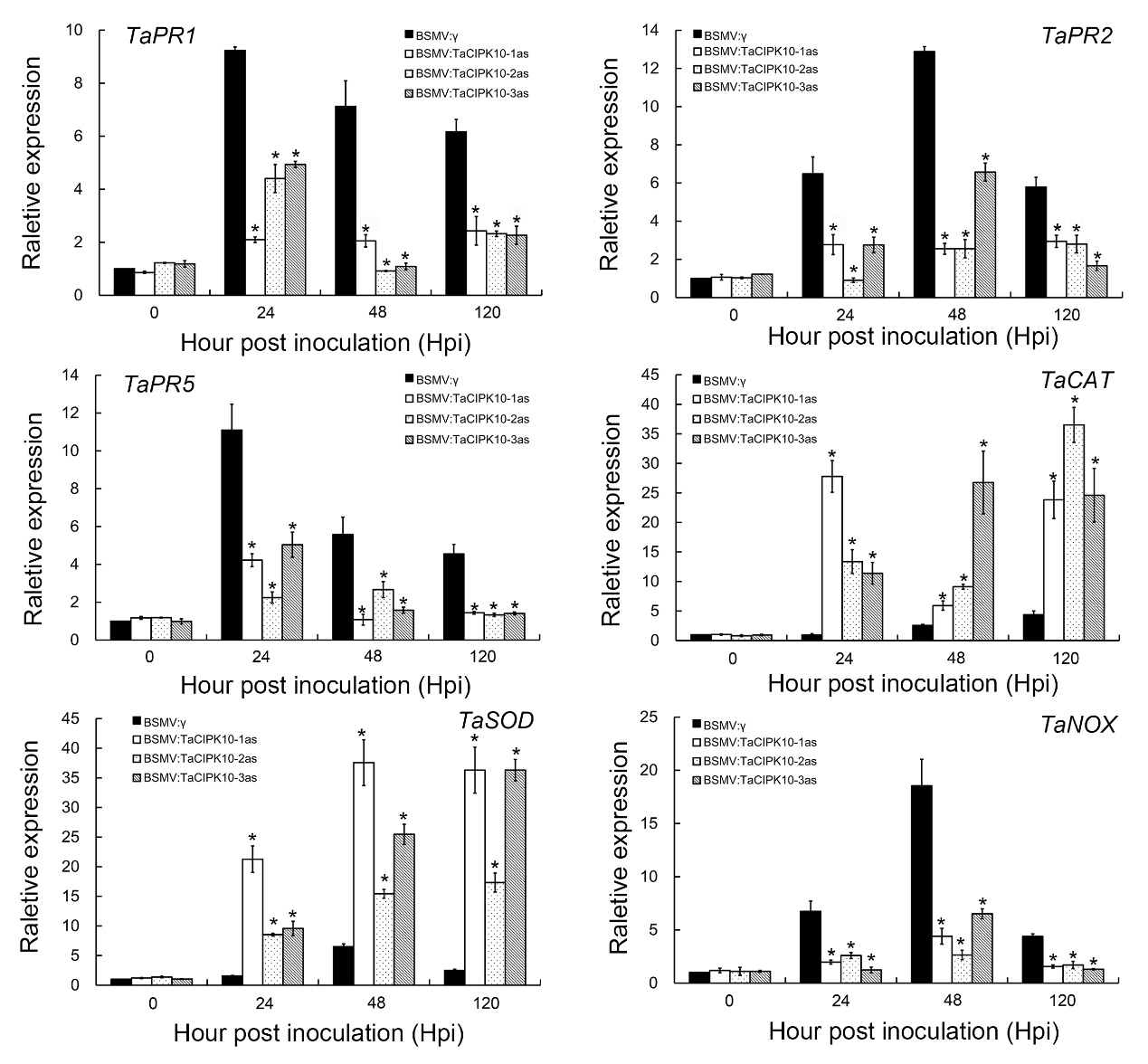


**Figure S8 Relative transcript levels of *TaPR1*, *TaPR2*, *TaPR5*, *TaSOD*, *TaCAT* and *TaNOX* in TaCIPK10-knockdown plants inoculated with CYR23.** The expression level was measured by qRT-PCR. The results were obtained from three biological replicates. The quantitative RT-PCR values were normalized to those for *TaEF-1α*, and are presented as fold changes relative that in plants with BSMV:γ treatment at time 0. The transcript level of genes in control plants at time 0 was standardized as 1. Data represent the mean of three biological replicates ± SE. Asterisks indicate significant differences between that in TaCIPK10-knockdown plants and control plants using Student t’ test (P < 0.01).


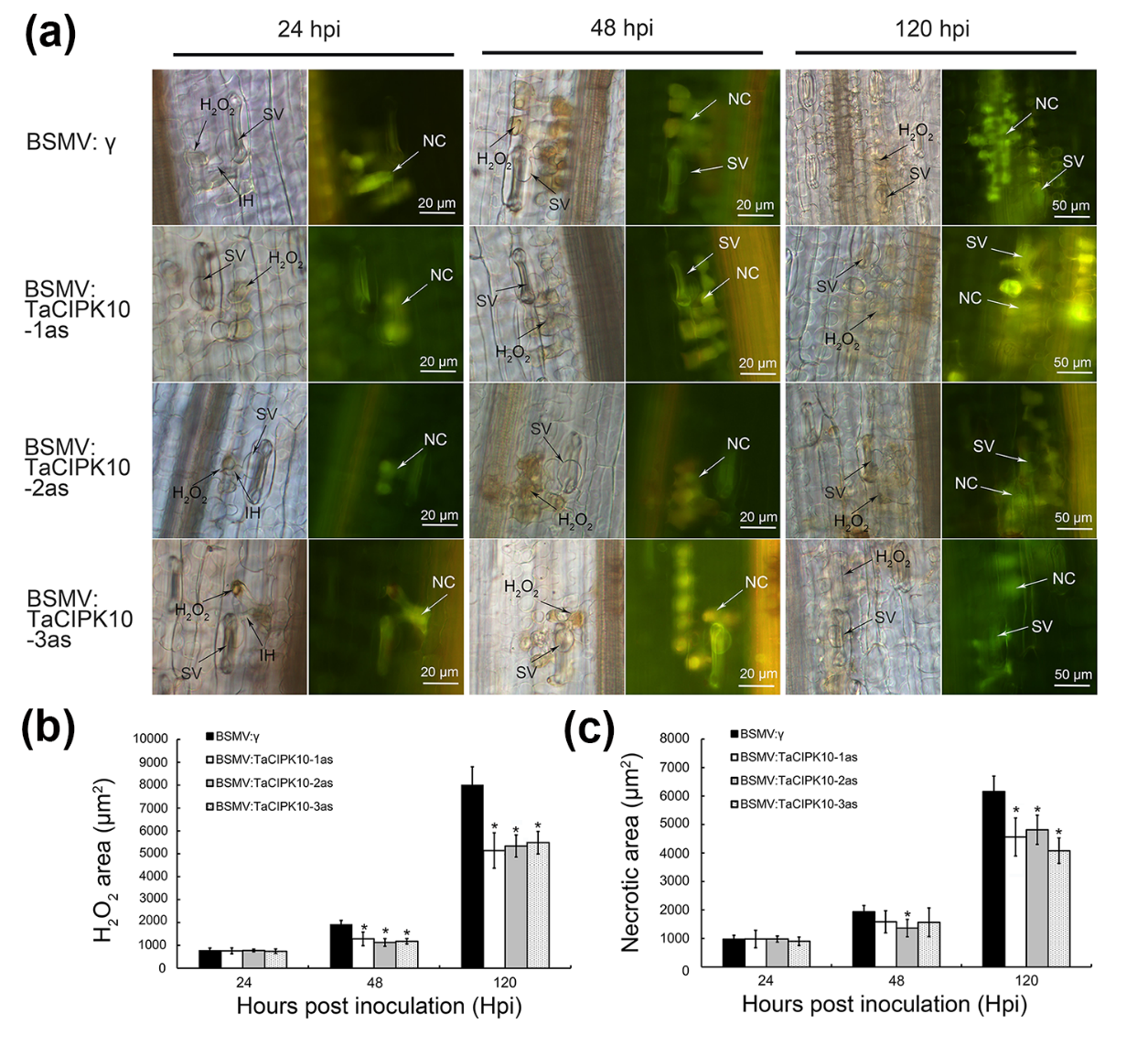


**Figure S9 Host response of TaCIPK10-knockdown plants inoculated with *Pst* avirulent race CYR23. a**, Histological observations of wheat leaves treated with BSMV and infected with CYR23. Wheat leaves which were pre-infected with BSMV:γ or BSMV:TaCIPK10-1/2/3as were subsequently inoculated with CYR23. 3,3-diaminobenzidine (DAB) was used to detect H_2_O_2_ viewed under differential interference contrast optics, and the necrosis was observed by autofluorescence. SV, sub-stomatal vesicle; NC, necrotic cell; bar, 20 μm. **b**, The amount of H_2_O_2_ production was measured by calculating the DAB-stained area at each infected site using the DP-BSW software. **c**, The area of autofluorescence was measured to determine necrotic cell death. Values represent mean ± standard errors of three independent assays. Asterisks indicate significant differences between that in TaCIPK10-knockdown plants and control plants at the same time points using Student t’ test (P < 0.01).


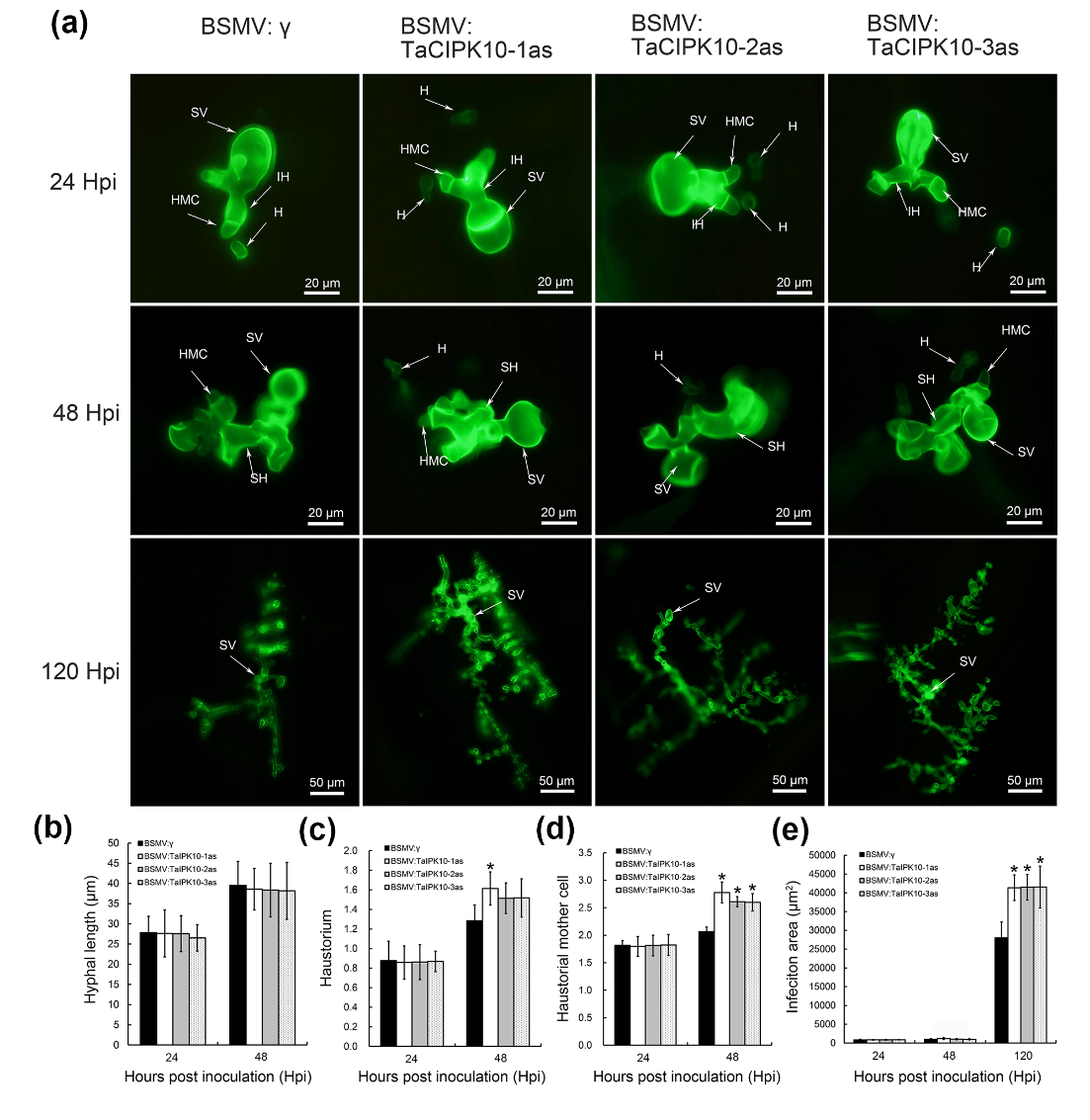


**Figure S10 Growth of *Pst* avirulent race, CYR23, was increased in TaCIPK10-knockdown plants. a**, The fungal structures were stained with wheat germ agglutinin (WGA) in wheat leaves inoculated with BSMV and Pst and observed under a fluorescence microscope. SV, sub-stomatal vesicle; HMC, haustorial mother cell; IH, infection hypha. H, haustoria. **b**, Hyphal length of CYR23, which is the average distance from the junction of the sub-stomatal vesicle and the hypha to the tip of the hypha, was measured using DP-BSW software (units in μm). **c** and **d**, The average of haustoria and haustorial mother cells in each infection site was determined. **e**, Infection area of CYR23, the average length of elongating hyphae, was calculated by DP-BSW software. All results were obtained from 50 infection sites and three biological replications were performed. Asterisks indicate a significant difference compared to that in BSMV: γ treatment plants at the same time points using Student t’ test (P < 0.01).


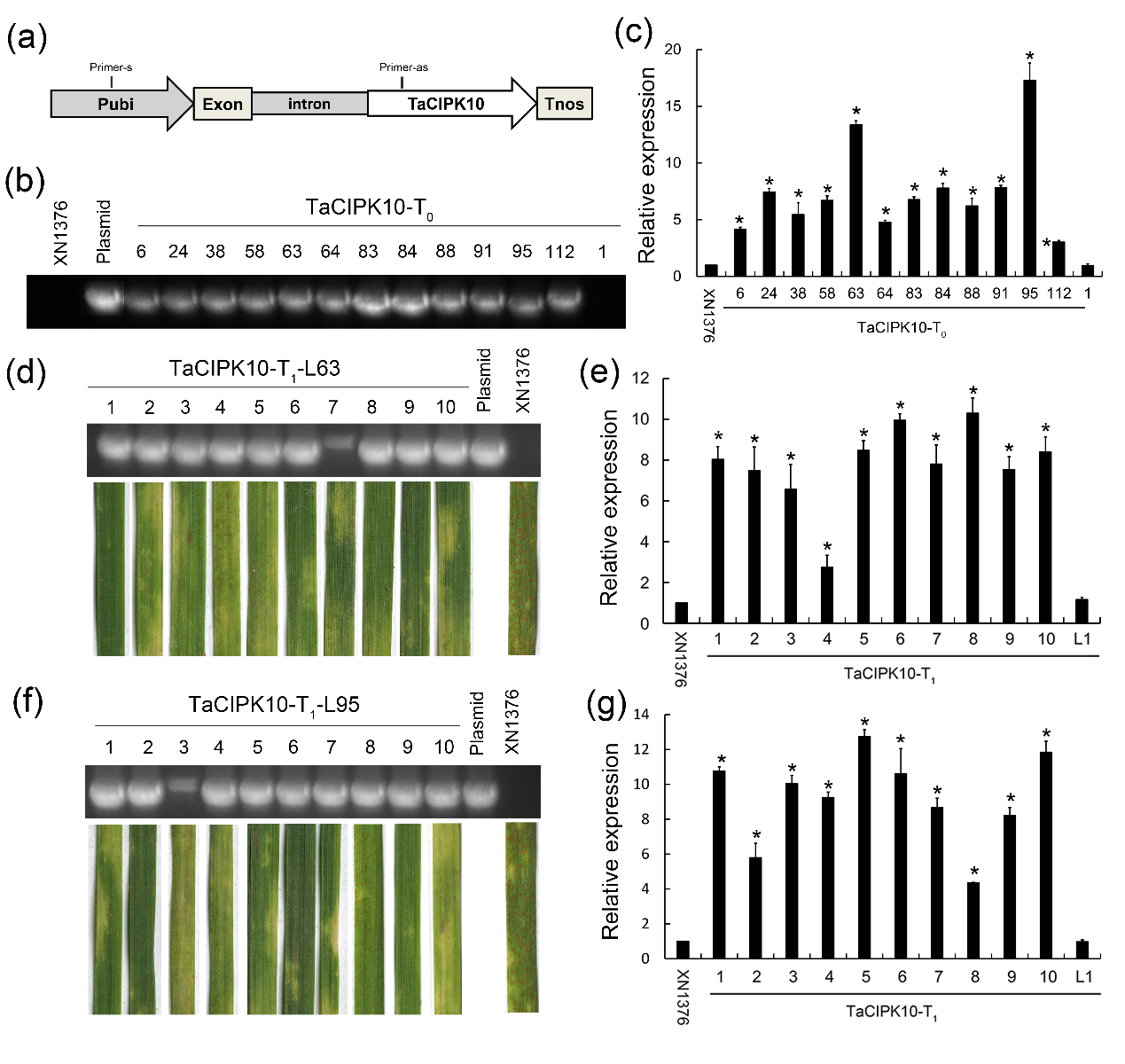


**Figure S11 Functional analysis of TaCIPK10 overexpression transgenic wheat. a,** Schematic representation of the transformation vector Pubi:TaCIPK10. TaCIPK10 in the transformation vector was driven by the Pubi promoter and terminated by Tnos. The superscripts indicate the primer sites for PCR assay. **b,** In T_0_ generation, 12 positive TaCIPK10 transgenic plants, TaCIPK10-T_0_-6, TaCIPK10-T_0_-24, TaCIPK10-T_0_-38, TaCIPK10-T_0_-58, TaCIPK10-T_0_-63, TaCIPK10-T_0_-64, TaCIPK10-T_0_-83, TaCIPK10-T_0_-84, TaCIPK10-T_0_-88, TaCIPK10-T_0_-91, TaCIPK10-T_0_-95, TaCIPK10-T_0_-112 were identified by PCR and TaCIPK10-T_0_-1 was negative control. **c**, The expression levels in the leaves of 12 positive transgenic plants, and one negative control (TaCIPK10-T_0_-112) were determined by qRT-PCR. XN1376 was used as control. **d** and **f**, PCR identification of two positive transgenic lines at T_1_ generation, TaCIPK10-T_1_-63 and TaCIPK10-T_1_-95. The positive transgenic line was inoculated with Pst race, CYR32, to evaluate wheat resistance. The wild type was used as the susceptible control. **e** and **g**, The expression levels of TaCIPK10 in the leaves of TaCIPK10-T_1_-63 and TaCIPK10-T_1_-95. The quantitative RT-PCR values were normalized to those for *TaEF-1α*, and are presented as fold changes relative to XN1376. The transcript level of TaCIPK10 in control plant was standardized as 1. Asterisks indicate significant differences between that in transformed and untransformed plants using student t’ test (P < 0.01).


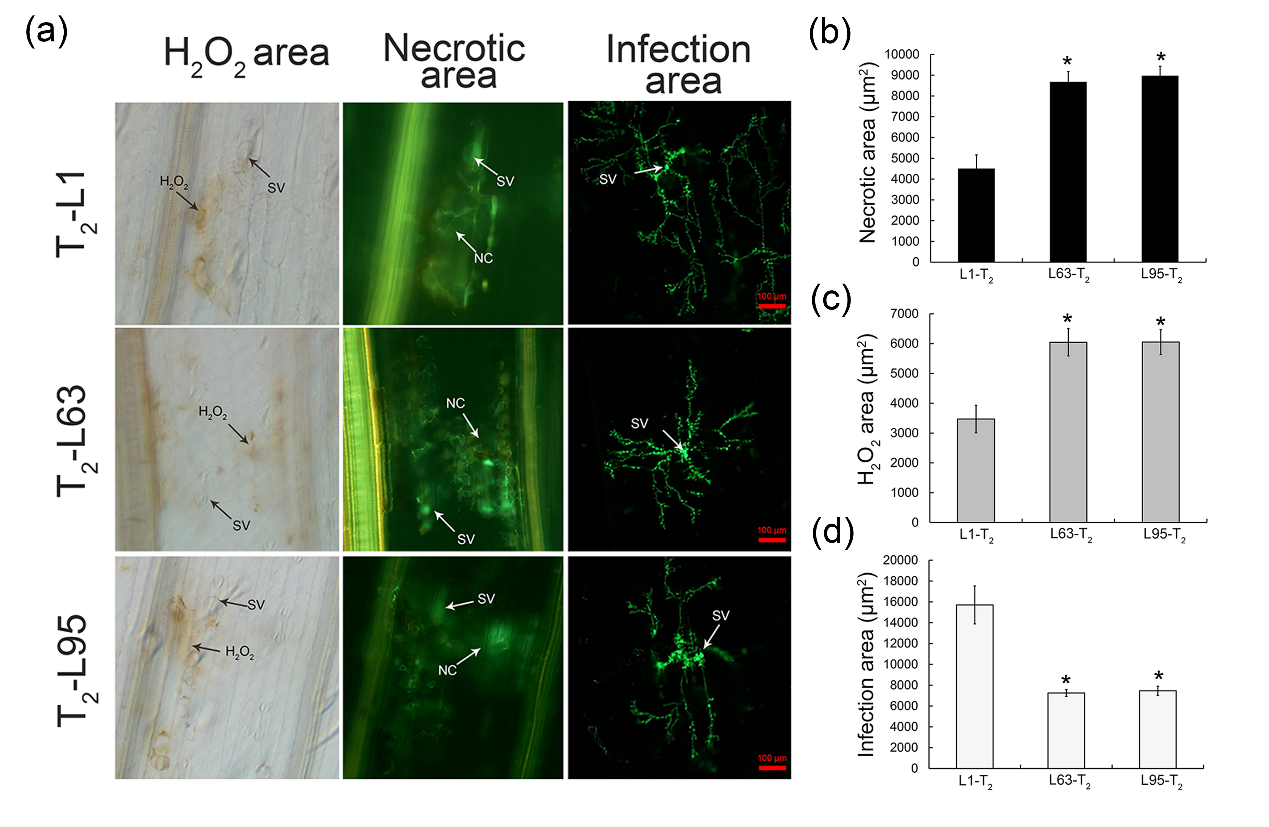


**Figure S12 Histological observation of positive *TaCIPK10* overexpression lines infected with CYR32 at 120 hpi. a,** H_2_O_2_ accumulation (first column) at infection sites was determined by staining with 3,3-diaminobenzidine (DAB) and viewed under differential interference contrast optics. The fungal structures were stained with wheat germ agglutinin (WGA) (third column) and observed under a fluorescence microscope. SV, sub-stomatal vesicle; NC, necrotic cell. Bar, 100 μm. **b**, The area of autofluorescence was measured to determine necrotic cell death at 5 hpi. **c,** The amount of H_2_O_2_ production was measured by calculating the DAB-stained area at each infection site using the DP-BSW software at 5 hpi. **d**, The infection area of CYR23 was calculated by DP-BSW software at 5 hpi. All results were obtained from 50 infection sites, and three biological replications were performed. Asterisks indicate significant differences between that in negative control and transformed plants using Student t’ test (P < 0.01).


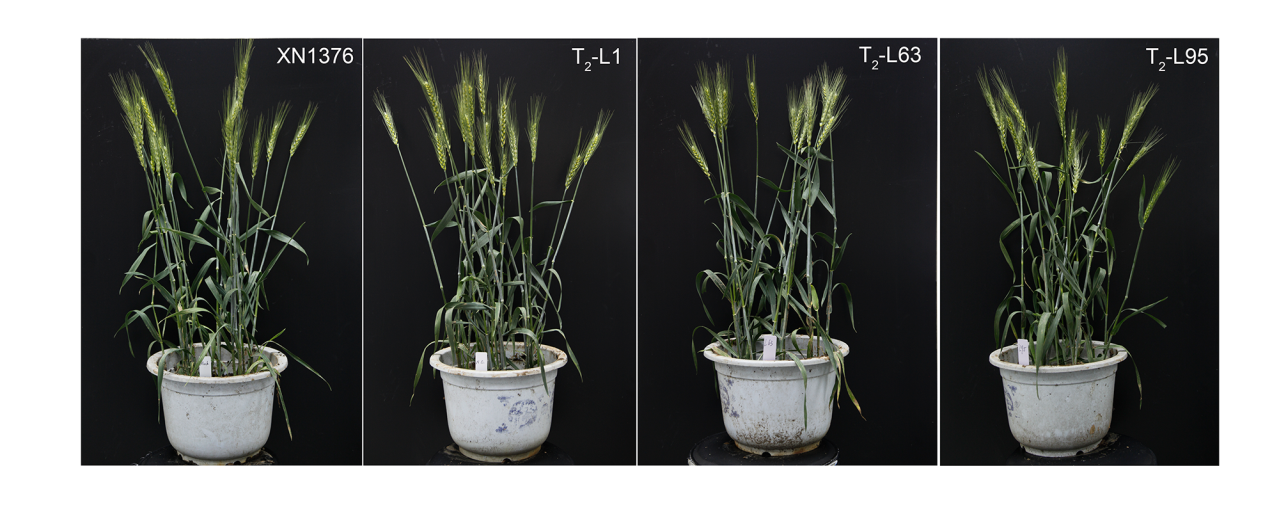


**Figure S13 TaCIPK10 overexpression transgenic lines did not affect the growth of wheat.**


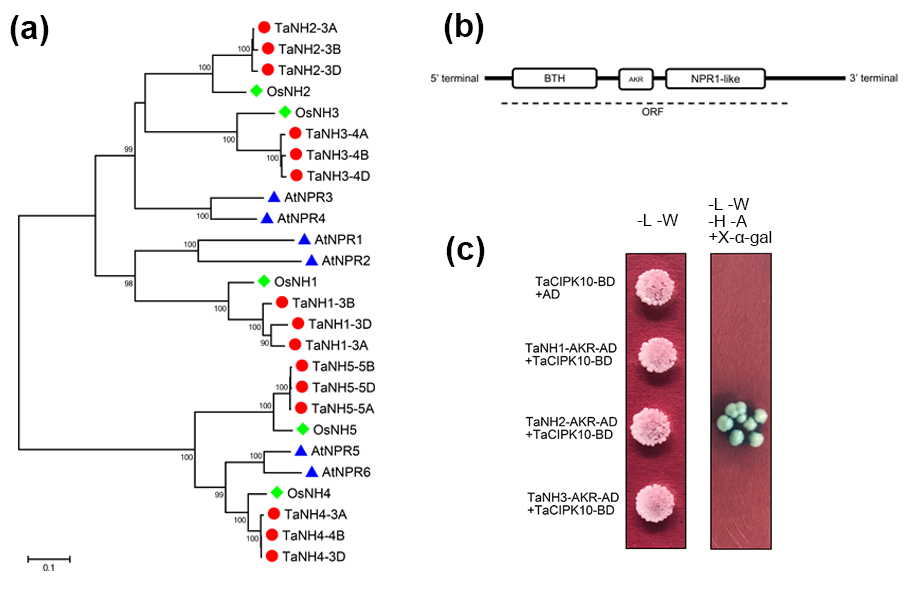


**Figure S14 TaCIPK10 interacted only with AKR motif of TaNH2. a**, Phylogenetic analysis of NPR family members from wheat, Arabidopsis and rice using MEGA 6.0 software. Ta, *Triticum aestivum*; Os, *Oryza sativa*; At, *Arabidopsis thaliana*. A, B, D in list of protein names represent the gene distribution in wheat chromosomes. The number above the internal branches indicates bootstrap values estimated based on 500 replications. **b**, Schematic structure of TaCIPK10 protein representing an ankyrin repeat (AKR) domain, a broad complex, tramtrack, and bric-à-brac/poxvirus and zinc-finger (BTB/POZ) domain and NPR1-like domain**. c**, Yeast two-hybrid analysis of interactions between TaCIPK10 and AKR motif of TaNH1, TaNH2 or TaNH3. Strain AH109 cultures containing TaCIPK10 in the GAL4 DNA-binding domain (BD) and AKR motif of TaNH1/2/3 in the GAL4 activation domain (AD) on nonselective (SD-LW) or selective (SD-LWHA) medium containing 20 μg/mL X-α-gal. The combination of AD and TaCIPK10-BD was used as the negative control.


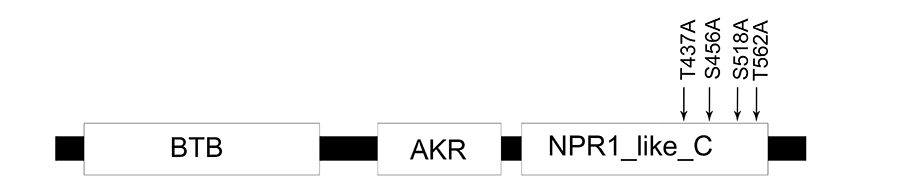


**Figure S15 Schematic diagrams of TaNH2.** The residues were predicted through PROSITE-based searches. The predicted serine (S) and threonine (T) residues were mutated to alanine (A) as indicated, resulting in a series of mutant proteins.


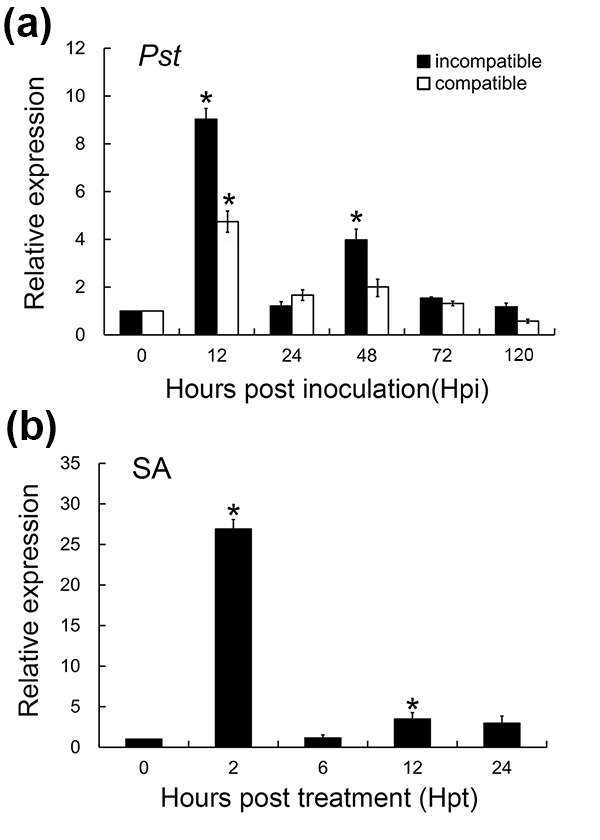


**Figure S16 TaNH2 was significantly induced by SA treatment and Pst inoculation. a**, Wheat leaves inoculated with Pst isolates CYR23 (incompatible interaction) and CYR31 (compatible interaction) were sampled at 0, 12, 24, 48, 72 and 120 hours post inoculation (hpi). **b**, Wheat seedlings were treated with 2 mM SA and sampled at 0, 2, 6, 12 and 24 hours post treatment (hpt). The quantitative RT-PCR values were normalized to those for *TaEF-1α* and are presented as fold changes relative that in plants without treatment at time 0. The transcript level of TaNH2 in the wheat leaves without treatment at time 0 was standardized as 1. Data are the means of three biological replicates ± SE. Asterisks indicate significant differences between time-course points using Student t’ test (P < 0.01).


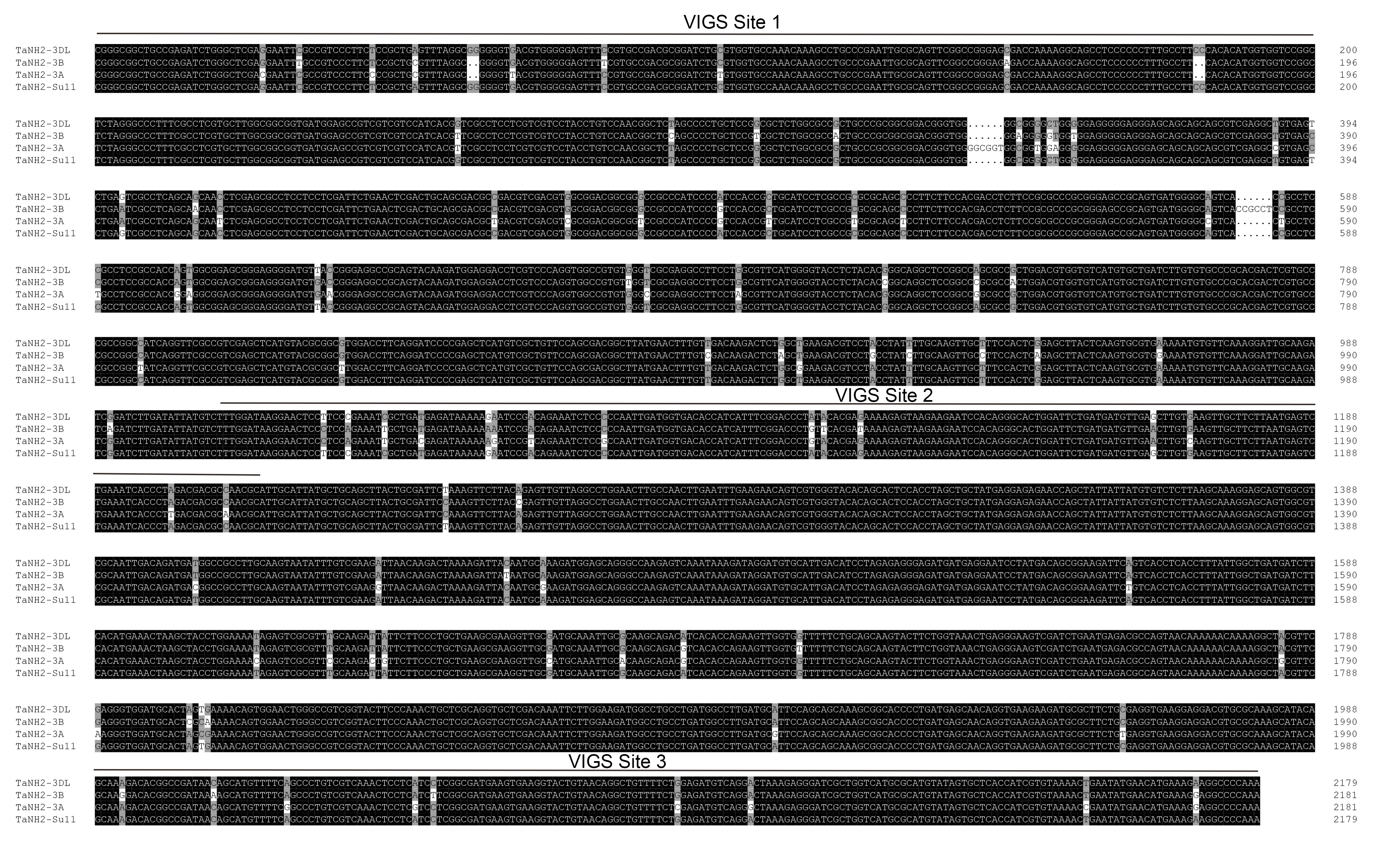


**Figure S17** **sequence alignment of the coding sequences for the three *TaNH2* copies in wheat genome database and TaNH2 cloned in Suwon 11.** VIGS sites 1 to 3 indicate the three VIGS sites of TaNH2, which are designated as TaNH2-1as, TaNH2-2as and TaNH2-3as, respectively.


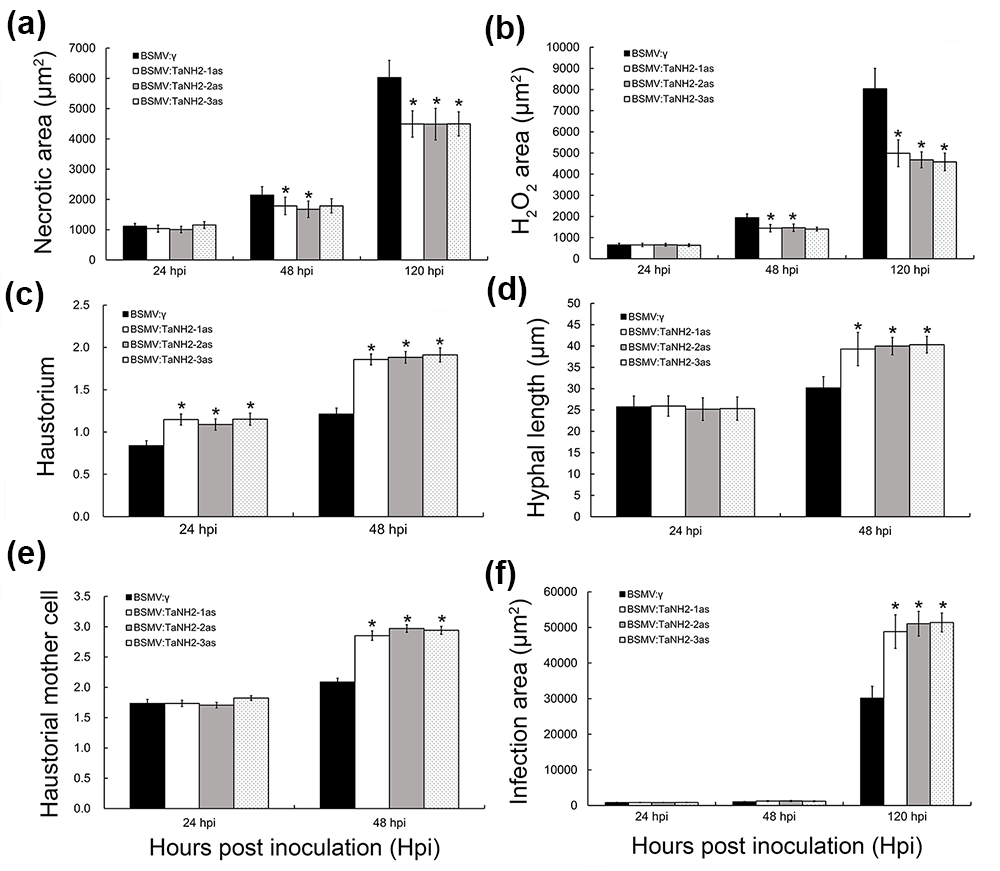


**Figure S18 Host response and histological observations of fungal growth in TaNH2-knockdown plants challenged by avirulent CYR23. a-d,** The number of haustoria or haustorial mother cells, hyphal length and the infection area were measured using DP-BSW software, respectively. **e**, The necrotic cell death was determined by the area of autofluorescence at each infection site. **f**, H_2_O_2_ accumulation was determined by calculating the DAB stained area at each infection site using the DP-BSW software. Values represent mean ± standard errors of three independent assays. All results were obtained from 50 infection sites, and three biological replications were included. Asterisks indicate significant differences between that in TaNH2-knockdown plants and control plants using Student t’ test (P < 0.01).


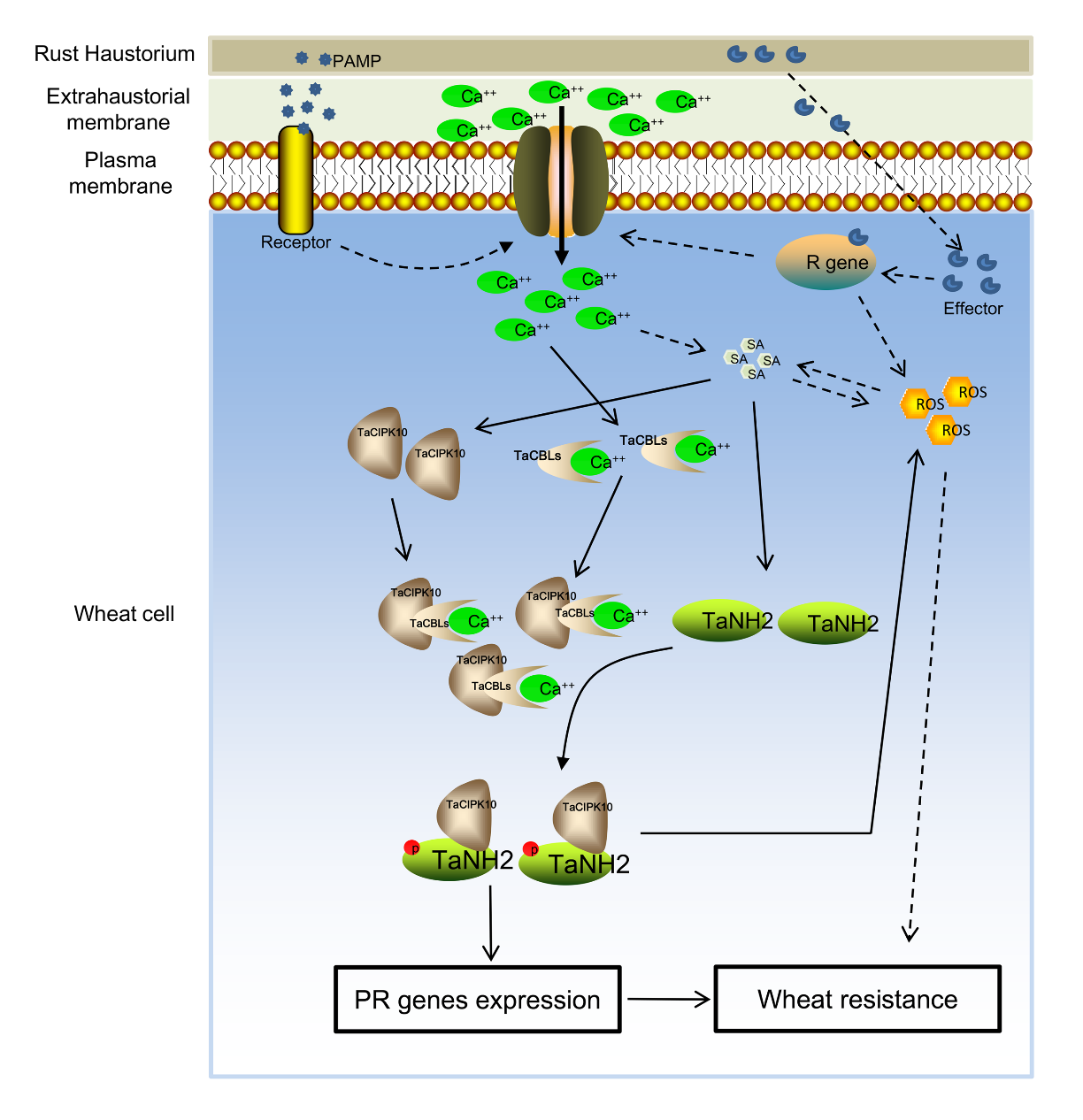


**Figure S19 Schematic presentation of possible molecular mechanism of *TaCIPK10*-mediated resistance processes relative to NPR1-like gene in wheat.** After *Pst* infection, the wheat immune system is activated, which stimulates the influx of Ca^2+^ from the intercellular space to the cytoplasm. The kinase activity of TaCIPK10 is activated by TaCBLs binding to Ca^2+^. Then, the activated TaCIPK10 is able to phosphorylate of TaNH2, which was the homologous of AtNPR3/4. TaNH2 and TaCIPK10 co-regulated wheat resistance to *Pst*. In addition, high concentration of Ca^2+^ enhances SA synthesis, which increases *TaNH2* and *TaCIPK10* expression. Black solid arrows indicate the results which were confirmed in this study. Black dotted arrows indicate the results which were confirmed in *Arabidopsis*.
