# Supplementary material for: TaCIPK10 interacts with and phosphorylates TaNH2 to activate wheat defense responses to stripe rust
Source: Plant Biotechnol J. 2018 Dec 5;17(5):956–68. doi: 10.1111/pbi.13031 (PMC6587807; doi:10.1111/pbi.13031)
Supplement: Supplementary file 2 — Table S1 Disease severity scale. Table S2 Primers used in this study. [file PBI-17-956-s001.pdf]

| Table S1 Disease Severity Scale | |
|---|---|
| Scale | Description |
| 0 | No Visable Infection |
| 1 | Necrotic/Chlorotic flecks without sporulation |
| 2 | Necrotic/Chlorotic stripes without sporulation |
| 3 | Necrotic/Chlorotic stripes with trace sporulation |
| 4 | Necrotic/Chlorotic stripes with light sporulation |
| 5 | Necrotic/Chlorotic stripes with intermediate sporulation |
| 6 | Chlorotic stripes with moderate sporulation |
| 7 | Chlorotic stripes with abundant sporulation |
| 8 | Stripes without chlorotic,  moderate sporulation |
| 9 | Stripes without chlorotic,  abundant sporulation |

**Table S2 Primers used in this study.**

| Function | name | sequence 5' to 3' |
|---|---|---|
| gene ampification | TaCIPK10-S | AATGCCGGAAGACGTGCTTGTG |
| | TaCIPK10-AS | TAAACTACCTACAAAAATTAGAC |
| | TaNH2-S | CGGGCGGCTGCCGAGATCT |
| | TaNH2-AS | TTTTGGGGCCTTCTTTCATGTTCA |
| qRT-PCR | QF-CIPK10 | AGACTTCACAGAGCGTTGCC |
| | QR-CIPK10 | TCTTTCAGCCTTCCTCGTTG |
| | QF-TaNH2 | GGGAGGGGATGTTACCG |
| | QR-TaNH2 | GAGCCTGCCCGTGTAGAG |
| | QF-TaCIPK05 | AGTTTCCCCCCCAATCACAG |
| | QR-TaCIPK05 | CCTTGCCCGAGCATACGAC |
| | QF-TaCIPK09 | GGATGGTAAAGCGGGAAAC |
| | QR-TaCIPK09 | GCACATTGAAACCAAGAGGG |
| | QF-TaCIPK10 | TAGAGGACTACCTTGGTTGGGA |
| | QR-TaCIPK10 | AGGCAGTGATGTTGTAAGTTGAG |
| | QF-TaCIPK14 | GCACCAGGGCATCTATCCA |
| | QR-TaCIPK14 | TTTGTCACCATTAGGGGCTTC |
| | QF-TaCIPK15 | CGCCATTCAGACTCCGCTAT |
| | QR-TaCIPK15 | TTCCCTAACAATCTCCCCACC |
| | QF-TaCIPK23 | AGAACAAACTACCACCAGACTCC |
| | QR-TaCIPK23 | AACCATTCAGCCAATACCCAC |
| | QF-TaPR1 | GAGAATGCAGACGCCCAAGC |
| | QR-TaPR1 | CTGGAGCTTGCAGTCGTTGATC |
| | QF-TaPR2 | AGGATGTTGCTTCCATGTTTGCCG |
| | QRTaPR2 | AAGTAGATGCGCATGCCGTTGATG |
| | QF-TaPR5 | CAAGCAGTGGTATCAACGCAGAG |
| | QR-TaPR5 | GTGAAGCCACAGTTGTTCTTGATGTT |
| | QF-TaSOD | CCGAGGTCTGGAACCATCAC |
| | QRTaSOD | AGCCGAAATCCTTCTCGATCT |
| | QF-TaCAT | TCGGACACCGAGGACCTATC |
| | QR-TaCAT | CCGTGCATGAACAACACGTT |
| | QF-TaNOX | ATGTTCGGCAACTTGGTGACT |
| | QR-TaNOX | CGTCTGCTCTAAGAAGACCACTTTT |
| | QF-TaEF | TGGTGTCATCAAGCCTGGTATGGT |
| | QR-TaEF | ACTCATGGTGCATCTCAACGGACT |
| | TaCIPK10(S)-32a-s | GTAACCTGAAAATCTCTGACTTCGGTCTGgacGCTCTGGC |
| | TaCIPK10(S)-32a-As | GCCAGAGCgtcCAGACCGAAGTCAGAGATTTTCAGGTTAC |
| | TaCIPK10(Y)-32a-s | GCTGCACACCACCTGCGGTACCCCGGCTgacGTTGCTCC |
| | TaCIPK10(Y)-32a-As | GGAGCAACgtcAGCCGGGGTACCGCAGGTGGTGTGCAGC |
| | TaCIPK10(T)-32a-As | ACCGCAGGTgtcGTGCAGCAGACCGTCCTGACGTTTGCA |
| | TaCIPK10(T)-32a-s | TGCAAACGTCAGGACGGTCTGCTGCACgacACCTGCGGT |
| | TaCIPK10(K)-32a-As | CGATAACgttGATAGCAACAGACTGAGAGGTTTTCA |
| | TaCIPK10(K)-32a-s | TGAAAACCTCTCAGTCTGTTGCTATCaacGTTATCG |
| | TaCIPK10-32a-S | CGGGATCCATGGTTGAAAAAAAAGGTAACAT |

| | | |
|---|---|---|
| protein expression | TaCIPK10-32a-AS | GGAATTCCGGCTGTTCACCCTGCCA |
| | TaCIPK10(Q)-32a-As | TTTCAGCAGACCACCGTCACGTTCGGTAGCTTCCTGGATG |
| | TaCIPK10(Q)-32a-s | CATCCAGGAAGCTACCGAACGTGACGGTGGTCTGCTG |
| | TaCBL6-32a-s | GTCGACAAATGGTGGATTTCCCGGAAGGG |
| | TaCBL6-32a-as | GCGGCCGCAAGCATCCTCGACCTGAGAGTT |
| | TaCBL4-Pro-s | CGGGATCCATGGGCTGCGTGTTGTCATC |
| | TaCBL4-Pro-as | CCGGAATTCTTTGCTGATTCCGCTGTAATC |
| | TaNH2-4T1-M1-AS | AGACGACGGCAGATGTTAGATTTTTCGTGGATCGGGTCAGAGAT |
| | TaNH2-4T1-M2-S | TCTGACCCGATCCACGAAAAATCTAACATCTGCCGTCGTCT |
| | TaNH2-4T1-S | CGGGATCCATGGAACCGTCTTCTTCTAT |
| | TaNH2-4T1-AS | GGAATTCTTTCATAGCAGAAGAAGAAGAGT |
| | TaNH2(AKR)-4t1-S | CGGGATCCCGTGTTCGTCGTATCCACCG |
| | TaNH2(AKR)-4t1-AS | GGAATTCAGCCAGACGACCGTCGTCGGTC |
| primers for yeast two hybrid | TaCIPK10-BD-S | CCGGAATTCATGGTAGAGAAGAAGGGAAAT |
| | TaCIPK10-BD-AS | TCCCCCGGGAACTAGGGCTGCTCACCTTGCCAAG |
| | TaNH2-AD-S | TCCCCCGGGATGGAGCCGTCGTCGTCC |
| | TaNH2-AD-AS | CTAGTCTAGAGCTTCATCGCCGAGGATGA |
| | TaCP10-AD(M)-S1 | GCTCTAGAGGATGGTAGAGAAGAAGGGAAATA |
| | TaCP10-AD(M)-AS1 | TTCAACAACCCACCGTCTCTGAGCTCTGTTGCTTCTTGTAT |
| | TaCP10-AD(M)-S2 | AATACAAGAAGCAACAGAGCTAGAGACGGTGGGTTGTTGAA |
| | TaCP10-AD(M)-AS2 | CCATCGATGGGCTGCTCACCTTGCCAA |
| | F-BD-TaCBL1.1 | CATGCCATGGGGTGCATCCAGTCCAC -3 ' |
| | R-BD-TaCBL1.1 | GGCTGCAGTCATGTAACAATATCATCAACCT |
| | TaCBL1.2-S | CATGCCATGGGGTGCTTCCACTCCATG |
| | TaCBL1.2-AS | GGCTGCAGCGTCACGATGTCGTCGAC |
| | F-BD-TaCBL2 | CATGCCATGGTGCAGTGTCTCGACGG |
| | R-BD-TaCBL2 | GGCTGCAGTCAGGTATCGTCGACCTGAGA |
| | F-BD-TaCBL3 | CATGCCATGGCAATGTTGCACTGCCTGGAGG |
| | R-BD-TaCBL3 | GGCTGCAGTTATGTATCGTCGACCTGAGAAT |
| | F-BD-TaCBL4 | CATGCCATGGGCTGCGTGTTGTCATC |
| | R-BD-TaCBL4 | GGCTGCAGTTATTTGCTGATTCCGCTGTAA |
| | F-BD-TaCBL6 | CATGCCATGGTGGATTTCCCGGAAGG |
| | R-BD-TaCBL6 | GGCTGCAGTCAAGCATCCTCGACCTGAGA |
| | F-BD-TaCBL9 | CATGCCATGGCCTCACGCTTCAGCTT |
| | R-BD-TaCBL9 | GGCTGCAGTTAGTCTTCAACCGCGGTATTG |
| | F-BD-TaNPR1(full) | GGAATTCATGGAGCCGTCGTCGTCCATCA |
| | R-BD-TaNPR1(full) | TCCCCCGGGTCACTTCATCGCCGAGGAT |
| | F-BD-TaNPR1(akr) | GGAATTCAAGAAGAATCCACAGGGCACT |
| | R-BD-TaNPR1(akr) | TCCCCCGGGCTTCATCGCCGAGGATGAGGA |
| | R-BD-TaNPR1(akr) | TCCCCCGGGCTTCATCGCCGAGGATGAGGA |
| | F-BD-TaNPR1(akr) | GGAATTCAAGAAGAATCCACAGGGCACT |
| | R-BD-TaNPR1(akr) | TCCCCCGGGCTTCATCGCCGAGGATGAGGA |
| | R-BD-TaNPR1(akr) | TCCCCCGGGCTTCATCGCCGAGGATGAGGA |
| | TaCP10-V2-S | CCTTAATTAACGGACAGGCCTTGACGAGGG |
| | TaCP10-V2-AS | AAGGAAAAAAGCGGCCGCTCCTCAAACATGCCAGAGAGAT |
| | TaCP10-V3-S | CCTTAATTAACATCTGGTGGAAATCAGGAAGA |

| | | |
|---|---|---|
| primer for VIGS | TaCP10-V3-AS | AAGGAAAAAAGCGGCCGCAGACAAGTGCATTATTGATTCC |
| | F-TaCIPK10-V | CCTTAATTAACGTGCTTGTGTGGTAGAGGAC |
| | R-TaCIPK10-V | TATGCGGCCGCGGCAGTGATGTTGTAAGTTGA |
| | TaNH2-V1-S1 | CCTTAATTAAATGGAGCCGTCGTCGTCC |
| | TaNH2-V1-AS1 | AAGGAAAAAAGCGGCCGCAATCGAGGAGGAGGCGCTCG |
| | F-TaNPR1-as1 | CCTTAATTAAACCATCATTTCGGACCCTAT |
| | R-TaNPR1-as1 | TATGCGGCCGCCCATCATCTGTCAATTGCG |
| | F-TaNPR1-as2 | CCTTAATTAAAAGAAGATGCGCTTCTGCG |
| | R-TaNPR1-as2 | TATGCGGCCGCCATCTTCTACTATTTTGGGG |
| primer for Co-IP | TaNH2(AKR)-TOPO-S | CACCCGTGTTCGTCGTATCCACCG |
| | TaNH2(AKR)-TOPO-AS | AGCCAGACGACCGTCGTCGGTCAGC |
| | TaNH2-TOPO-S | CACCATGGAGCCGTCGTCGTCCAT |
| | TaNH2-TOPO-AS | CTTCATCGCCGAGGATGAGG |
| | TaCP10-TOPO-S | CACCATGGTAGAGAAGAAGGGAAATATC |
| | TaCP10-TOPO-AS | CTAGGGCTGCTCACCTTGC |
| primer for overexpression | TaCP10-Pubi-F2 | AAACGCCGTCGACGAGTCTAAC |
| | TaCP10-OE-R1 | GCCCCAGCAGTCTTCCCAT |
| | TaCIPK10-003-s3 | TCCCCCGGGATGGTAGAGAAGAAGGGAAAT |
| | TaCP10-003-AS | TCCCCCGGGCTAGGGCTGCTCACCTTGCCA |
